# A Body Shape Index mediates the associations of geriatric nutritional risk index with activities of daily living disability in an older rural population in Guangxi, China

**DOI:** 10.3389/fpubh.2026.1808377

**Published:** 2026-04-24

**Authors:** Wenjie Liang, Haiyan Lu, Kaiyong Huang, Li Yang

**Affiliations:** 1 Department of Social Medicine, School of Public Health, Guangxi Medical University, Nanning, Guangxi, China; 2 Department of Occupational and Environmental Health, School of Public Health, Guangxi Medical University, Nanning, Guangxi, China

**Keywords:** A Body Shape Index, activities of daily living, cross-sectional study, disability, geriatric nutritional risk index, mediation analysis, older adults, rural population

## Abstract

**Background:**

Malnutrition and disability in Activities of Daily Living (ADL) are significant health concerns among the older adults. The Geriatric Nutritional Risk Index (GNRI) is a validated nutritional assessment tool; however, the mechanisms linking it to ADL disability, particularly in rural settings, remain incompletely understood. This study investigated the associations between GNRI and Basic ADL (BADL), Instrumental ADL (IADL), and overall ADL disability, and assessed the potential mediating roles of biomarkers related to inflammation, metabolism, obesity, and insulin resistance.

**Methods:**

This cross-sectional study (2016–2018) included 4,399 community-dwelling adults aged ≥60 years from Donglan County, Guangxi, China. BADL and IADL were assessed using the Chinese-adapted versions of the Physical Self-Maintenance Scale (PSMS) and the Lawton Instrumental Activities of Daily Living Scale, respectively. GNRI was calculated and categorized as normal (>98) or low (≤98). Biomarkers included the Systemic Inflammation Response Index (SIRI), Aggregate Index of Systemic Inflammation (AISI), Metabolic Score for Insulin Resistance (METS-IR), A Body Shape Index (ABSI), and Triglyceride-Glucose Index (TyG). Associations were examined using multivariable logistic and generalized linear models. Restricted cubic splines explored nonlinearity, and mediation analyses quantified indirect effects. Subgroup and sensitivity analyses were conducted. Statistical analyses were performed using IBM SPSS Statistics (version 26.0) and Zstats (version 1.0).

**Results:**

In fully adjusted models, low GNRI was associated with higher odds of BADL (OR = 2.10, 95% CI: 1.56–2.83), IADL (OR = 1.69, 95% CI: 1.34–2.12), and ADL (OR = 2.18, 95% CI: 1.60–2.97) disability. GNRI was negatively associated with ABSI (*β* = −0.0017, *p* < 0.001), which was positively associated with all three disability types (ORs: 1.45–2.00). RCS analyses indicated nonlinear relationships for GNRI. ABSI significantly mediated 24.39 to 31.07% of the associations between GNRI and disability. The GNRI-disability association was significantly stronger in participants with anemia (P for interaction <0.001). Sensitivity analyses confirmed the robustness of these findings.

**Conclusion:**

Lower GNRI is independently associated with increased risks of BADL, IADL, and ADL disability in the rural Chinese older adult population. ABSI mediates a substantial portion of these associations, suggesting that malnutrition may influence functional disability partly through adverse body shape changes, such as central obesity. These findings highlight the potential value of integrated nutritional and body composition management, especially for anemic older adults. From a public health perspective, routine screening for nutritional risk using GNRI, combined with central obesity assessment, should be considered in rural primary care settings. Future longitudinal studies and intervention trials are needed to establish causality and evaluate the effectiveness of targeted nutritional and body composition interventions in preventing or delaying ADL disability.

## 1 Introduction

Global population aging poses a significant public health challenge, making the preservation of functional independence and quality of life in older adults a paramount goal (1). Disability in Activities of Daily Living (ADL), a core marker of functional decline, is strongly associated with increased morbidity, mortality, and healthcare burdens (2, 3). This challenge is particularly acute in resource-limited rural settings, where older adults often face compounded disadvantages such as reduced access to healthcare, socioeconomic constraints, and weaker community support systems (4, 5).

Malnutrition is a highly prevalent yet frequently under-diagnosed condition among the older adults, serving as a key contributor to sarcopenia, immune dysfunction, and adverse health outcomes (6). The Geriatric Nutritional Risk Index (GNRI), a simple and objective tool derived from serum albumin and body weight, has proven effective in predicting clinical outcomes such as mortality and complications in hospitalized older populations (7, 8). However, evidence linking GNRI to ADL disability in community-dwelling older adults, especially within large-scale rural Chinese populations, remains insufficient and is often limited by small sample sizes or a focus on specific clinical subgroups (9, 10). More critically, the biological pathways underlying this association are not well delineated.

Accumulating evidence suggests that GNRI may influence physical function through interconnected biological pathways involving chronic low-grade inflammation, metabolic dysregulation, and unfavorable body composition (11, 12). Malnutrition often coexists with a pro-inflammatory state. Composite indices like the Systemic Inflammation Response Index (SIRI) and the Aggregate Index of Systemic Inflammation (AISI) reflect overall inflammatory burden and are linked to functional decline (13, 14). Concurrently, malnutrition is intertwined with insulin resistance---assessable via indices like the Triglyceride-Glucose Index (TyG) and Metabolic Score for Insulin Resistance (METS-IR)---and with detrimental shifts in body composition (15, 16). Notably, central obesity, a condition characterized by excessive visceral fat, is independently associated with both metabolic risk and functional impairment (17, 18). The A Body Shape Index (ABSI), which adjusts waist circumference for height and body mass index (BMI), is a specific indicator of central adiposity and associated health risks (19). While previous studies have separately examined links between nutrition and inflammation, metabolism, or body composition, research that systematically evaluates whether these factors mediate the relationship between GNRI and ADL disability is scarce. This gap is especially prominent in rural community-dwelling older adult population (20).

Therefore, utilizing cross-sectional data from an older adult population in rural Southwest China, this study aimed to investigate the associations between GNRI and ADL disability (encompassing both basic and instrumental ADL). Furthermore, we sought to examine the potential mediating roles of biomarkers representing systemic inflammation (SIRI, AISI), insulin resistance (TyG, METS-IR), and central obesity (ABSI) in these associations.

## 2 Patients and methods

### 2.1 Study site and participants

This cross-sectional survey was conducted between August 2016 and July 2018 in Donglan County, Hongshuihe Basin, Guangxi Zhuang Autonomous Region, Southwest China. Potential participants were identified through village registration records maintained by local community health centers. Community health workers and village leaders assisted in approaching eligible individuals.

Inclusion criteria were: (a) age ≥60 years at enrollment, and (b) residence in the study townships for ≥10 years.

Exclusion criteria were: (a) diagnosis of neurological/psychiatric disorders (e.g., Alzheimer’s disease, dementia, cognitive impairment); (b) severe sensory impairments (blindness, deafness, or mutism); and (c) active malignancy.

**Figure 1:**
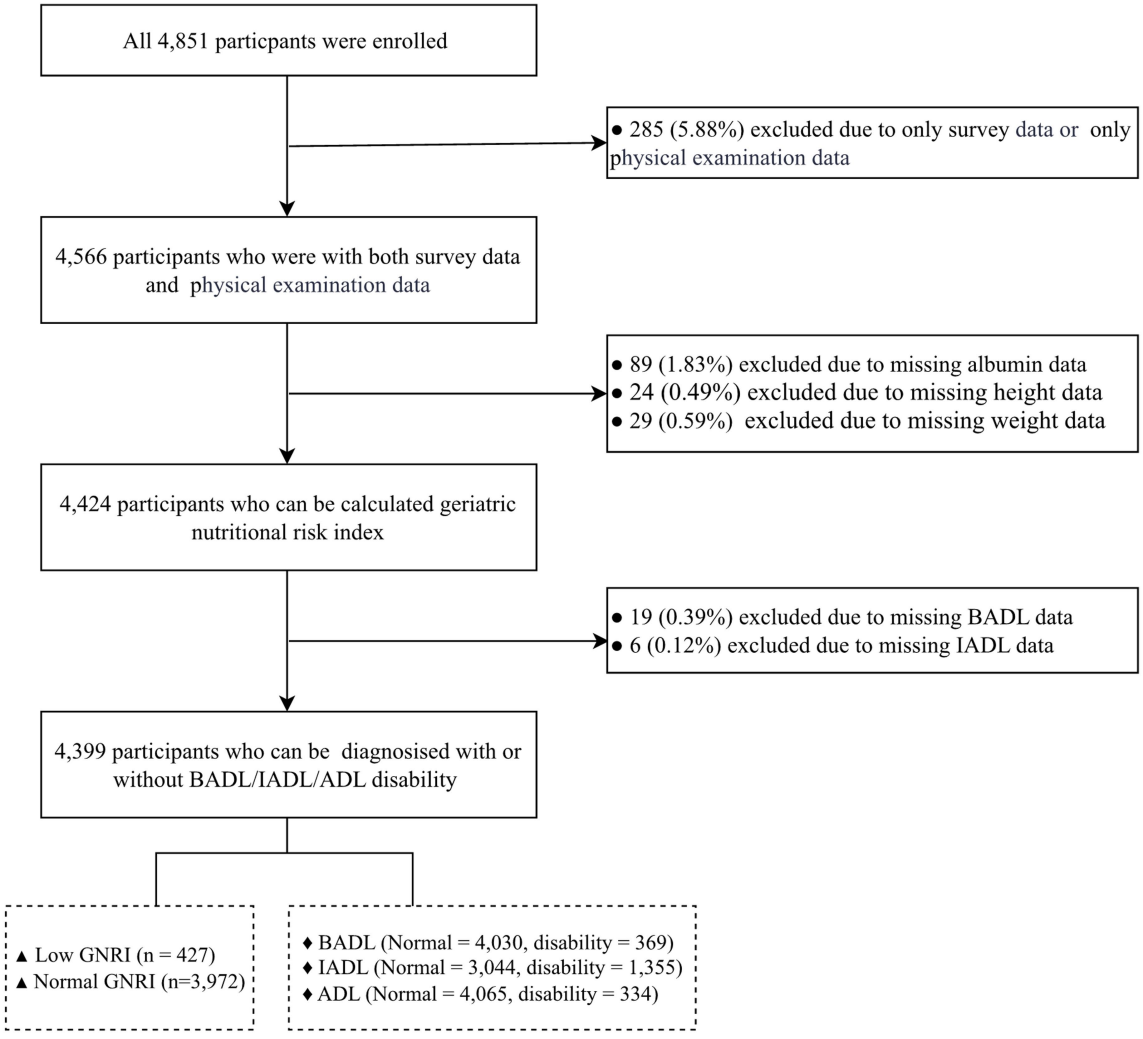
Flow diagram for participants in the study. BADL, basic activities of daily living; IADL, instrumental activities of daily living; ADL, activities of daily living; GNRI, geriatric nutritional risk index.

A total of 4,851 individuals were initially enrolled. After excluding participants with: (1) missing questionnaire or physical examination data (*n* = 285); (2) missing data required for Geriatric Nutritional Risk Index (GNRI) calculation (*n* = 142); (3) missing Activities of Daily Living (ADL) data (*n* = 25), a final sample of 4,399 participants was included in the analysis (Figure 1).

### 2.2 Data collection

Designated local clinics across 8 townships in Donglan County served as data collection sites, ensuring that participants traveled no more than 30 min to reach a site. The data collection team comprised 12 trained investigators, including 4 geriatric medicine physicians, 4 public health graduate students, and 4 community health nurses. All investigators completed a standardized 3-day training program prior to data collection, covering study objectives and ethical protocols, administration of structured questionnaires, standardized anthropometric measurements, blood sample collection and handling procedures, and quality control protocols. Inter-rater reliability was assessed for anthropometric measurements, with intra-class correlation coefficients >0.90 achieved before field data collection commenced.

Each participant visit lasted approximately 90–120 min and included: a structured interview (45–60 min) collecting sociodemographic characteristics, health history, and ADL assessments; a physical examination (20–30 min) including anthropometric measurements and blood pressure; blood sample collection (10 min) of fasting venous blood; and a rest period with health education (15 min).

### 2.3 Ethical considerations

The study protocol was approved by the Ethics Committee of Guangxi Medical University (approval number: 201503010-2). All procedures performed were in accordance with the ethical standards of the institutional research committee and with the 1964 Helsinki declaration and its later amendments. Written informed consent was obtained from all participants prior to data collection. Participant confidentiality was protected through multiple measures, including the use of unique study identifiers, separate storage of linking files, confidentiality training for data collectors, secure storage of paper forms, and reporting of results in aggregate form only. During data collection, participants with abnormal findings were referred to local hospitals for further evaluation, and no adverse events occurred.

### 2.4 Outcome variables

Functional ability was assessed using Chinese-adapted versions of the Physical Self-Maintenance Scale (for BADL) and the Lawton Instrumental Activities of Daily Living Scale (for IADL) (21, 22). The BADL scale included six items: walking, feeding, dressing, grooming, bathing, and toileting. The IADL scale included eight items: using transportation, food preparation, housekeeping, managing medications, shopping, laundry, telephone use, and handling finances. Both scales used a four-point scoring system: (1) “Independent without difficulty,” (2) “Independent with some difficulty,” (3) “Requires partial assistance,” and (4) “Completely dependent.” A score ≥2 on any item indicated functional limitation (10). Operational definitions were: BADL disability (limitation in ≥1 BADL item); IADL disability (limitation in ≥1 IADL item); ADL disability (concurrent limitation in ≥1 BADL and ≥1 IADL item), dichotomized as “no disability” vs. “disability.” Psychometric analysis showed strong internal consistency: Cronbach’s *α* was 0.946 for the BADL scale and 0.837 for the IADL scale.

### 2.5 Data collection instruments and measures

#### 2.5.1 Sociodemographic and health questionnaire

A structured questionnaire was developed to collect sociodemographic characteristics and health-related information. Items included: age (in years), sex (male/female), marital status (single/partnered), educational attainment (no formal education/primary school or above), annual household income (<10,000 RMB/≥10,000 RMB), smoking status (never/former/current), alcohol consumption (never/former/current), and self-reported physician-diagnosed chronic conditions (cerebrovascular disease, rheumatism, osteoarthritis, hypertension, diabetes). The questionnaire was developed based on a literature review and standardized questionnaires used in previous geriatric epidemiological studies in China. Content validity was assessed by a panel of five experts, and the questionnaire was pilot-tested with 50 community-dwelling older adults (not included in the main study), leading to minor wording modifications.

#### 2.5.2 Geriatric nutritional risk index (GNRI)

GNRI was calculated using the formula developed by Bouillanne et al. (23):


$$\text{GNRI}=[1.489\times \text{albumin}\;(g/L)]+[41.7\times (\begin{array}{l} \text{actual weight}\\ /\text{ideal weight} \end{array})]$$

Ideal weight was derived from the Lorentz equations:

Men: Height (cm) – 100 – [(Height (cm)–150)/4]
Women: Height (cm) – 100 – [(Height (cm) – 150)/2.5]

If the actual weight exceeded ideal weight (ratio ≥1), the ratio was set to 1 to avoid overestimation of nutritional risk. Participants were categorized as normal nutritional status (GNRI >98) or low nutritional status (GNRI ≤98) based on established cutoffs (23). Serum albumin was measured using the bromocresol green method (coefficient of variation <3%).

#### 2.5.3 Biomarker assessment

Fasting venous blood samples were collected between 7:00–9:00 a.m. after an overnight fast (≥8 h). Complete blood counts were analyzed using an automated hematology analyzer (Sysmex XN-9000, Japan). Biochemical parameters (fasting plasma glucose, triglycerides, HDL-C, albumin) were measured using an automated chemistry analyzer (Hitachi 7,600–020, Japan). All assays were performed at the Central Laboratory of Guangxi Medical University following standardized protocols with internal and external quality control.

Five composite biomarkers were calculated:

Systemic Inflammation Response Index (SIRI) = Neutrophil count (10^9^/L) × Monocyte count (10^9^/L)/Lymphocyte count (10^9^/L) (24)
Aggregate Index of Systemic Inflammation (AISI) = (Neutrophil count × Monocyte count × Platelet count)/Lymphocyte count (25)
Metabolic Score for Insulin Resistance (METS-IR) = Ln[2 × FPG (mg/dL) + TG (mg/dL)] × BMI/Ln[HDL-C (mg/dL)] (26)
A Body Shape Index (ABSI) = Waist circumference (m)/[BMI^(2/3) × Height (m)^(1/2)] (19)
Triglyceride-Glucose Index (TyG) = Ln[Fasting TG (mg/dL) × FPG (mg/dL)/2] (16)

#### 2.5.4 Anthropometric measurements

Anthropometric measurements were performed by trained investigators using standardized techniques. Height was measured to the nearest 0.1 cm using a wall-mounted stadiometer (Seca, Germany) with participants standing barefoot. Weight was measured to the nearest 0.1 kg using a calibrated electronic scale (Tanita, Japan) with participants wearing light clothing. Waist circumference was measured at the midpoint between the lower rib margin and the iliac crest using a non-stretchable tape measure. BMI was calculated as weight (kg) divided by height squared (m^2^). All measurements were taken twice, and the average was used for analysis.

### 2.6 Quality control measures

Several quality control measures were implemented: (1) All questionnaires were reviewed on-site by field supervisors for completeness and consistency; (2) A random 10% of participants were re-interviewed within one week by a different investigator to assess response consistency (kappa coefficients >0.85 for all key variables); (3) Anthropometric equipment was calibrated daily using standard procedures; (4) Blood samples were processed within 4 h of collection and stored at −80 °C until analysis; (5) Double data entry was performed with range and logic checks to minimize data entry errors.

### 2.7 Statistical analysis

Statistical analyses were performed using IBM SPSS Statistics Version 26.0 (IBM Corp., Armonk, NY, United States) and Zstats software.^1^ A two-tailed *α* level of 0.05 was set as the threshold for statistical significance, with *p* < 0.05 considered statistically significant. The specific analytical methods are described as follows:

#### 2.7.1 Descriptive statistics

The Kolmogorov–Smirnov test was used to assess the normality of continuous variables. Normally distributed continuous variables are presented as mean ± standard deviation (mean ± SD), and categorical variables are expressed as frequencies (percentages) [*n*(%)]. The chi-square test was employed to compare differences in categorical variables between groups stratified by Geriatric Nutritional Risk Index (GNRI).

#### 2.7.2 Association analyses

Binary logistic regression models were used to estimate the odds ratios (ORs) and 95% confidence intervals (95% CIs) for two types of associations: (1) associations between GNRI and various types of disabilities (BADL, IADL, and ADL disabilities); (2) associations between biomarkers (dichotomized by the median) and various types of disabilities.

Generalized linear models were applied to analyze the associations between continuous GNRI and continuous biomarkers, with *β* coefficients and 95% CIs indicating the strength of associations.

Four sequentially adjusted models were constructed:

Model 1 (unadjusted model): no covariates adjusted
Model 2 (basic adjusted model): adjusted for age, sex, marital status, educational attainment, and annual household income
Model 3 (multivariate adjusted model): further adjusted for cerebrovascular disease, osteoarthropathy, rheumatism, hypertension, diabetes, estimated glomerular filtration rate (eGFR), anemia, alanine transaminase (ALT), and aspartate transaminase (AST) on the basis of Model 2
Model 4 (fully adjusted model): additionally adjusted for smoking status and alcohol consumption on the basis of Model 3

#### 2.7.3 Model fit and multicollinearity tests

The Omnibus test (*p* < 0.05 indicating the overall statistical significance of the model) and Hosmer-Lemeshow goodness-of-fit test (*p* > 0.05 indicating a good model fit) were used to evaluate the fitting effect of the regression models. Multicollinearity was assessed using variance inflation factors (VIFs), with VIF < 5 suggesting no significant multicollinearity.

#### 2.7.4 Nonlinear relationship analysis

Restricted Cubic Spline (RCS) models were used to explore the exposure-response relationships between GNRI and various types of disabilities, with the number of knots determined by minimizing the Akaike Information Criterion (AIC).

#### 2.7.5 Mediation analysis

Mediation analysis was performed to quantify the direct and indirect effects, with GNRI as the independent variable (X), biomarkers as the mediating variables (M), and various types of disabilities as the dependent variables (Y) (27).

#### 2.7.6 Subgroup and interaction analyses

Stratified subgroup analyses were conducted by age (60–69 years/≥70 years), sex (male/female), hypertension status (presence/absence), and anemia status (presence/absence). Interaction terms were introduced to test the interaction effects between GNRI and the aforementioned stratifying variables in the associations with disabilities.

#### 2.7.7 Sensitivity analyses

Four methods were used to verify the robustness of the results: (1) Repeating the analyses with different covariate adjustment combinations; (2) Repeating the association analyses after reclassifying GNRI into quartiles; (3) Using modified Poisson regression to estimate prevalence ratios (PRs) instead of ORs; (4) Repeating the analyses after excluding participants with metabolic syndrome.

#### 2.7.8 Covariate and missing data handling

Potential confounders included age (60–69 years, ≥70 years), sex, marital status (single, partnered), educational attainment (<primary school, ≥primary school), annual household income (<10,000 CNY, ≥10,000 CNY), cerebrovascular disease, rheumatism, osteoarthropathy, hypertension, diabetes, anemia, eGFR (normal, reduced), AST (normal, elevated), ALT (normal, elevated), smoking status, and alcohol consumption. Detailed information is provided in Supplementary Table S1.

The proportion of missing data for all covariates was <5% (Supplementary Table S2). Missing values were coded as “unknown” in regression analyses without imputation.

## 3 Results

### 3.1 Baseline characteristics of the study participants

A total of 4,851 individuals were initially enrolled in the study. After excluding participants who did not meet the eligibility criteria, the final analytical sample consisted of 4,399 participants. The prevalences of BADL disability, IADL disability, and ADL disability were 8.39, 30.80, and 7.59%, respectively.

As summarized in Table 1, the mean (SD) age of the participants was 69.95 (7.22) years. The majority of participants were aged 60–69 years, female, living with a partner, had less than primary school education, an annual household income ≥10,000 RMB, and were free from major chronic conditions (cerebrovascular disease, osteoarthropathy, rheumatism, hypertension, and diabetes). Most participants had preserved renal function (eGFR ≥60 mL/min/1.73m^2^), were non-anemic, had normal liver enzyme levels, and were non-smokers and non-drinkers. Overall, 90.29% of participants had a normal GNRI, while 9.71% were classified as having a low GNRI.

**Table 1:** General characteristics of the study participants according to geriatric nutritional risk index.

Variables	Total *n*(%)	GNRI *n*(%)		*p* value
		Normal	Low	
Total	4,399 (100.00)	3,972 (90.29)	427 (9.71)	
Sex				0.852
Male	1774 (40.33)	1,600 (40.28)	174 (40.75)	
Female	2,625 (59.67)	2,372 (59.72)	253 (59.25)	
Age group (years)				**<0.001**
60–69	2,343 (53.26)	2,192 (55.19)	151 (35.36)	
≥ 70	2056 (46.74)	1780 (44.81)	276 (64.64)	
Marital status				**<0.001**
Partnered	3,050 (69.44)	2,800 (70.60)	250 (58.69)	
Single	1,342 (30.56)	1,166 (29.40)	176 (41.31)	
Educational attainment				**0.020**
Less than primary school	2022 (46.07)	1803 (45.50)	219 (51.41)	
Primary school and above	2,367 (53.93)	2,160 (54.50)	207 (48.59)	
Annual income				**<0.001**
<10,000 RMB	1,664 (38.03)	1,468 (37.29)	196 (46.67)	
≥ 10,000 RMB	2,712 (61.97)	2,469 (62.71)	243 (53.33)	
Cerebrovascular disease				0.753
No	4,196 (95.39)	3,790 (95.42)	406 (95.08)	
Yes	203 (4.61)	182 (4.58)	21 (4.92)	
Osteoarthropathy				0.817
No	3,735 (84.92)	3,374 (84.97)	361 (84.54)	
Yes	663 (15.08)	597 (15.03)	66 (15.46)	
Rheumatism				0.598
No	3,899 (88.69)	3,517 (88.61)	382 (89.46)	
Yes	497 (11.31)	452 (11.39)	45 (10.54)	
Hypertension				**<0.001**
No	2034 (46.26)	1784 (44.93)	250 (58.69)	
Yes	2,363 (53.74)	2,187 (55.07)	176 (41.31)	
Diabetes				0.922
No	3,921 (89.13)	3,541 (89.15)	380 (88.99)	
Yes	478 (10.87)	431 (10.85)	47 (11.01)	
eGFR (mL/min/1.73m^2^)				**<0.001**
≥ 60	3,758 (86.23)	3,422 (86.94)	336 (79.62)	
<60	600 (13.77)	514 (13.06)	86 (20.38)	
Anemia				**<0.001**
No	2,809 (64.04)	2,632 (66.43)	177 (41.75)	
Yes	1,577 (35.96)	1,330 (33.57)	247 (58.25)	
ALT				0.829
Normal	3,450 (78.43)	3,128 (78.75)	322 (75.41)	
High	949 (21.57)	844 (21.25)	105 (24.59)	
AST				0.111
Normal	4,152 (94.39)	3,748 (94.36)	404 (94.61)	
High	247 (5.61)	224 (5.64)	23 (5.39)	
Smoking status				0.295
No	3,706 (84.34)	3,355 (84.53)	351 (82.59)	
Yes	688 (15.66)	614 (15.47)	74 (17.41)	
Alcohol consumption				0.355
No	3,352 (76.60)	3,018 (76.41)	334 (78.40)	
Yes	1,024 (23.40)	932 (23.59)	92 (21.60)	

GNRI, geriatric nutritional risk index; RMB, renminbi; eGFR, estimated glomerular filtration rate; AST, aspartate aminotransferase; ALT, alanine aminotransferase. Bold text in the table represents statistically significant results.

Participants with a low GNRI were more likely to be older, single, have lower educational attainment and annual household income, be non-hypertensive, have reduced renal function, and suffer from anemia. The proportion of missing covariate data was minimal (<5%), with detailed information provided in Supplementary Table S1.

### 3.2 Associations between GNRI and ADL disabilities

The results of the logistic regression analyses are presented in Table 2. In the fully adjusted model (Model 4), participants with a low GNRI had a significantly higher risk of BADL disability (OR = 2.10; 95% CI: 1.56–2.83), IADL disability (OR = 1.69; 95% CI: 1.34–2.12), and ADL disability (OR = 2.18; 95% CI: 1.60–2.97) compared to those with a normal GNRI. Variance inflation factors (VIFs) for all models were below 5, indicating no significant multicollinearity (Supplementary Table S2). The robustness of the models was confirmed by statistically significant Omnibus tests (all *p* < 0.05) and non-significant Hosmer-Lemeshow goodness-of-fit tests (all *p* > 0.05).

**Table 2:** Associations of GNRI with BADL, IADL, and ADL disability.

Models	GNRI, OR (95%CI)		*p* value
	Normal	Low	
BADL disability			
Model 1	1.00 (Ref.)	2.67 (2.02, 3.51)	<0.001
Model 2	1.00 (Ref.)	2.15 (1.61, 2.86)	<0.001
Model 3	1.00 (Ref.)	2.10 (1.56, 2.83)	<0.001
Model 4	1.00 (Ref.)	2.10 (1.56, 2.83)	<0.001
IADL disability			
Model 1	1.00 (Ref.)	2.06 (1.68, 2.52)	<0.001
Model 2	1.00 (Ref.)	1.71 (1.37, 2.13)	<0.001
Model 3	1.00 (Ref.)	1.67 (1.33, 2.09)	<0.001
Model 4	1.00 (Ref.)	1.69 (1.34, 2.12)	<0.001
ADL disability			
Model 1	1.00 (Ref.)	2.87 (2.17, 3.81)	<0.001
Model 2	1.00 (Ref.)	2.28 (1.70, 3.05)	<0.001
Model 3	1.00 (Ref.)	2.19 (1.61, 2.97)	<0.001
Model 4	1.00 (Ref.)	2.18 (1.60, 2.97)	<0.001

GNRI, geriatric nutritional risk index; BADL, basic activities of daily living; IADL, instrumental activities of daily living; ADL, activities of daily living; OR, odds ratio. Model 1 was unadjusted model; Model 2 adjusted for sex, age, marital status, educational attainment, annual income; Model 3 further adjusted cerebrovascular disease, rheumatism, osteoarthropathy, hypertension, diabetes, estimated glomerular filtration rate, anemia, alanine aminotransferase, aspartate aminotransferase; Model 4 further adjusted smoking status and alcohol consumption.

### 3.3 Association of GNRI with biomarkers

Generalized linear model analyses (Table 3) revealed that in the fully adjusted model, continuous GNRI was significantly positively associated with METS-IR (*β* = 0.0037, 95% CI: 0.0028–0.0044) and TyG (β = 0.013, 95% CI: 0.010–0.016). Conversely, GNRI was significantly negatively associated with ABSI (β = −0.0017, 95% CI: −0.0021 to −0.0014). No significant associations were found between GNRI and SIRI or AISI.

**Table 3:** Associations of GNRI with SIRI, AISI, METS–IR, ABSI, and TyG.

Indicators	Model 1		Model 2		Model 3		Model 4	
	β (95% CI)	*p* value	β (95% CI)	*p* value	β (95% CI)	*p* value	β (95% CI)	*p* value
SIRI	−0.001 (−0.006, 0.005)	0.836	0.001 (−0.006, 0.006)	0.915	−0.001 (−0.007, 0.005)	0.628	−0.002 (−0.008, 0.005)	0.625
AISI	−0.561 (−2.713, 1.592)	0.610	−0.412 (−2.623, 1.800)	0.715	−0.718 (−2.994, 1.558)	0.536	−0.735 (−3.024, 1.554)	0.529
METS–IR	0.0048 (0.0041, 0.0056)	<0.001	0.0047 (0.0039, 0.0055)	<0.001	0.0036 (0.0028, 0.0044)	<0.001	0.0037 (0.0028, 0.0044)	<0.001
ABSI	−0.0021 (−0.0024, −0.0017)	<0.001	−0.0017 (−0.0019, −0.0014)	<0.001	−0.0017 (−0.0020, −0.0014)	<0.001	−0.0017 (−0.0021, −0.0014)	<0.001
TyG	0.020 (0.017, 0.023)	<0.001	0.020 (0.017, 0.023)	<0.001	0.013 (0.010, 0.016)	<0.001	0.013 (0.010, 0.016)	<0.001

GNRI, geriatric nutritional risk index; SIRI, systemic inflammation response index; AISI, aggregate index of systemic inflammation; METS–IR, metabolic score for insulin resistance; ABSI, A Body Shape Index; TyG, triglyceride–glucose index; CI, confidence interval. Model 1 was unadjusted model; Model 2 adjusted for sex, age, marital status, educational attainment, annual income; Model 3 further adjusted cerebrovascular disease, rheumatism, osteoarthropathy, hypertension, diabetes, estimated glomerular filtration rate, anemia, alanine, aminotransferase, aspartate aminotransferase; Model 4 further adjusted smoking status and alcohol consumption.

### 3.4 Association of biomarkers with ADL disabilities

As shown in Table 4, after full adjustment and dichotomization at the median, only a high ABSI level was consistently and significantly associated with increased odds of all three types of disability: BADL (OR = 1.98; 95% CI: 1.55–2.52), IADL (OR = 1.45; 95% CI: 1.25–1.67), and ADL (OR = 2.00; 95% CI: 1.55–2.59).

**Table 4:** Associations of SIRI, AISI, METS–IR, ABSI, and TyG with BADL, IADL, and ADL disability.

Mediators	Model 1		Model 2		Model 3		Model 4	
	OR (95% CI)	*p* value	OR (95% CI)	*p* value	OR (95% CI)	*p* value	OR (95% CI)	*p* value
BADL disability								
SIRI	1.18 (0.95, 1.46)	0.130	1.07 (0.86, 1.34)	0.529	1.06 (0.85, 1.33)	0.594	1.05 (0.84, 1.31)	0.680
AISI	1.19 (0.96, 1.47)	0.114	1.14 (0.92, 1.42)	0.232	1.12 (0.90, 1.40)	0.317	1.11 (0.89, 1.38)	0.367
METS–IR	1.27 (1.02, 1.57)	0.031	1.31 (1.05, 1.63)	0.018	1.25 (0.99, 1.57)	0.060	1.22 (0.97, 1.54)	0.094
ABSI	**2.31 (1.83, 2.90)**	**<0.001**	**2.09 (1.65, 2.66)**	**<0.001**	**1.97 (1.55, 2.51)**	**<0.001**	**1.98 (1.55, 2.52)**	**<0.001**
TyG	1.10 (0.89, 1.36)	0.392	1.17 (0.94, 1.46)	0.160	1.08 (0.85, 1.37)	0.550	1.09 (0.86, 1.38)	0.500
IADL disability								
SIRI	1.07 (0.94, 1.21)	0.328	1.07 (0.93, 1.23)	0.357	1.07 (0.93, 1.23)	0.323	1.07 (0.93, 1.23)	0.345
AISI	1.01 (0.89, 1.14)	0.922	0.98 (0.85, 1.13)	0.777	0.99 (0.86, 1.13)	0.827	0.98 (0.85, 1.13)	0.804
METS–IR	0.82 (0.72, 0.93)	0.002	0.88 (0.77, 1.01)	0.077	0.89 (0.77, 1.02)	0.097	0.87 (0.75, 1.01)	0.059
ABSI	**1.83 (1.60, 2.08)**	**<0.001**	**1.47 (1.28, 1.70)**	**<0.001**	**1.43 (1.24, 1.65)**	**<0.001**	**1.45 (1.25, 1.67)**	**<0.001**
TyG	0.90 (0.79, 1.02)	0.091	0.94 (0.82, 1.08)	0.364	0.95 (0.82, 1.11)	0.520	0.96 (0.83, 1.11)	0.572
ADL disability								
SIRI	1.21 (0.97, 1.52)	0.095	1.10 (0.87, 1.38)	0.421	1.07 (0.85, 1.35)	0.566	1.06 (0.84, 1.33)	0.652
AISI	1.20 (0.96, 1.50)	0.110	1.15 (0.92, 1.45)	0.224	1.11 (0.88, 1.41)	0.363	1.10 (0.87, 1.39)	0.418
METS–IR	1.23 (0.98, 1.54)	0.072	1.27 (1.01, 1.61)	0.040	1.21 (0.95, 1.54)	0.124	1.18 (0.93, 1.50)	0.185
ABSI	**2.37 (1.86, 3.02)**	**<0.001**	**2.11 (1.64, 2.71)**	**<0.001**	**2.00 (1.55, 2.58)**	**<0.001**	2.00 (1.55, 2.59)	**<0.001**
TyG	1.06 (0.85, 1.33)	0.602	1.14 (0.90, 1.43)	0.281	1.03 (0.80, 1.32)	0.826	1.04 (0.81, 1.34)	0.766

SIRI, systemic inflammation response index; AISI, aggregate index of systemic inflammation; METS–IR, metabolic score for insulin resistance; ABSI, A Body Shape Index; TyG, triglyceride–glucose index; BADL, basic activities of daily living; IADL, instrumental activities of daily living; ADL, activities of daily living; OR, odds ratio; CI, confidence interval. Bold values indicate statistically significant associations (*p* < 0.05).

### 3.5 Nonlinear relationship analyses

**Figure 2:**
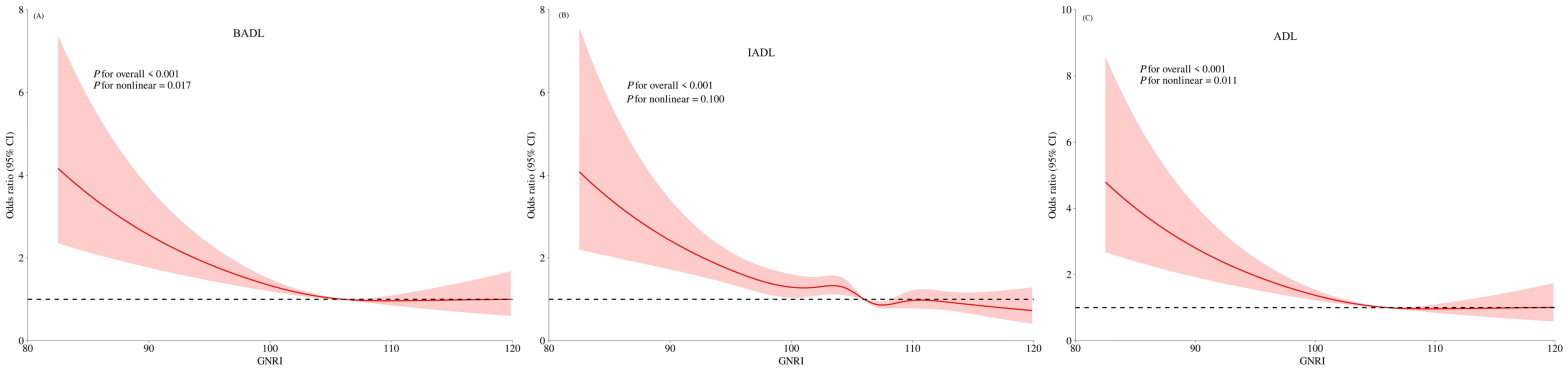
The non–linear relationships of GNRI with BADL, IADL, and ADL disability. **(A)** BADL disability, **(B)** IADL disability, **(C)** ADL disability. GNRI, geriatric nutritional risk index; BADL, basic activities of daily living; IADL, instrumental activities of daily living; ADL, activities of daily living. The models were adjusted for sex, age, marital status, educational attainment, annual income, cerebrovascular disease, rheumatism, osteoarthropathy, hypertension, diabetes, estimated glomerular filtration rate, anemia, alanine, aminotransferase, aspartate aminotransferase, smoking status, and alcohol consumption.

Restricted Cubic Spline (RCS) analyses visualized the exposure-response relationships (Figure 2). GNRI exhibited significant nonlinear, inverse associations with the risk of BADL, IADL, and ADL disability (P for nonlinear < 0.05 for all), with the risk increasing more sharply below specific GNRI thresholds.

Further RCS analyses of the biomarkers (Supplementary Figure S1) revealed distinct patterns: For BADL disability, METS-IR and TyG showed linear associations (P for nonlinear = 0.099 and 0.057), while ABSI exhibited a nonlinear relationship (P for nonlinear < 0.001). For IADL and ADL disability, METS-IR demonstrated linear associations (P for nonlinear = 0.072 and 0.06), whereas ABSI again showed significant nonlinearity (P for nonlinear < 0.001).

In addition, nonlinear relationships were observed between GNRI and AISI, METS–IR, ABSI, and TyG (Supplementary Figure S2).

### 3.6 Mediation effects of biomarkers

**Figure 3:**
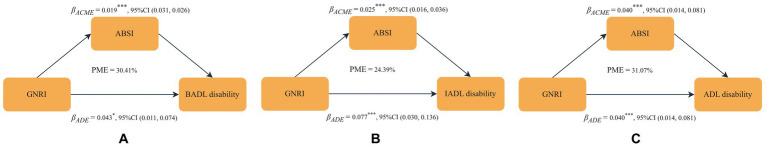
The mediation effects of ABSI in the relationships of GNRI with risks of BADL, IADL, and ADL disability. **(A)** BADL disability, **(B)** IADL disability, **(C)** ADL disability. ABSI, A Body Shape Index; BADL, basic activities of daily living; IADL, instrumental activities of daily living; ADL, activities of daily living. The models were adjusted for sex, age, marital status, educational attainment, annual income, cerebrovascular disease, rheumatism, osteoarthropathy, hypertension, diabetes, estimated glomerular filtration rate, anemia, alanine, aminotransferase, aspartate aminotransferase, smoking status, and alcohol consumption.

Mediation analyses were conducted to disentangle direct and indirect pathways (Figure 3; Supplementary Table S4). In the fully adjusted models, ABSI was identified as a significant mediator. It accounted for 30.41% of the association between GNRI and BADL disability, 24.39% for IADL disability, and 31.07% for ADL disability (all *p* < 0.001).

### 3.7 Subgroup and interaction analyses

**Figure 4:**
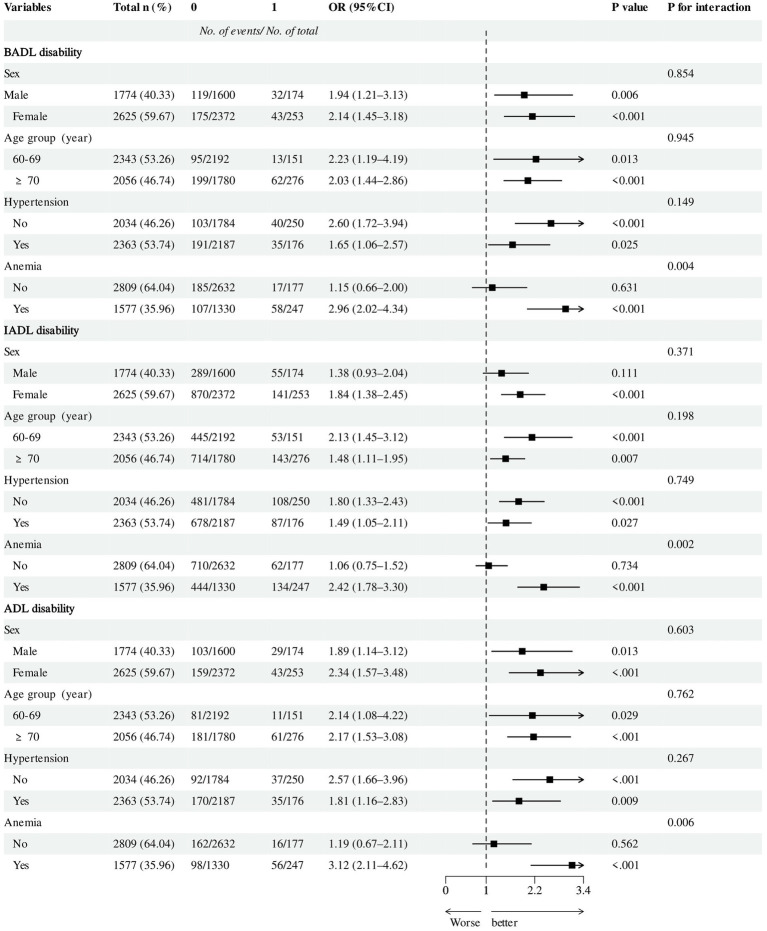
Subgroup analysis of associations of GNRI with BADL, IADL, and ADL disability. GNRI, geriatric nutritional risk index; BADL, basic activities of daily living; IADL, instrumental activities of daily living; ADL, activities of daily living; OR, odds ratio; CI, confidence interval. The models were adjusted for sex, age, marital status, educational attainment, annual income, cerebrovascular disease, rheumatism, osteoarthropathy, hypertension, diabetes, estimated glomerular filtration rate, anemia, alanine, aminotransferase, aspartate aminotransferase, smoking status, and alcohol consumption.

Subgroup analyses stratified by age, sex, hypertension, and anemia status are presented in Figure 4. The positive association between low GNRI and ADL disabilities was consistent across most subgroups. Interaction analysis confirmed a significant interaction between GNRI and anemia status for all three types of disability (all P for interaction < 0.001). The positive association was more pronounced in individuals with anemia.

Additional stratified mediation analyses (Supplementary Tables S4–S7) suggested that the strength of the ABSI-mediated pathway varied across subgroups. The mediated association was generally more pronounced in individuals aged ≥70 years, those with hypertension, and those without anemia.

### 3.8 Sensitivity analyses

The robustness of the primary findings was confirmed through multiple sensitivity analyses: (1) Categorizing GNRI into quartiles yielded consistent dose–response relationships with disability risks (Supplementary Table S8) and similar associations with biomarkers (Supplementary Table S9). (2) Using Modified Poisson regression to estimate prevalence ratios (PRs) instead of ORs produced virtually identical conclusions regarding the associations of GNRI (Supplementary Table S10) and biomarkers (Supplementary Table S11) with disabilities. (3) Excluding participants with metabolic syndrome did not materially alter the main associations between GNRI and disabilities (Supplementary Table S12), the observed nonlinear relationships (Supplementary Figures S3, S4), or the significant mediating role of ABSI, although the effect sizes were slightly attenuated (Supplementary Table S13).

## 4 Discussion

This study systematically examined the associations between GNRI and different dimensions of ADL disability, along with the underlying mediating mechanisms, in a rural Chinese older adult population. The key findings are that a lower GNRI is independently associated with an increased risk of both BADL and IADL disability, and that these associations are significantly mediated by ABSI, a marker of central obesity. Notably, the association between GNRI and BADL disability exhibited a nonlinear pattern, and the strength of GNRI’s association with both disability types significantly differed by anemia status. These findings deepen our understanding of the potential pathways linking malnutrition to functional decline and suggest potential targets for intervention.

### 4.1 Association between GNRI and BADL/IADL disability

This study demonstrates that lower GNRI is independently associated with both BADL and IADL disability in this rural Chinese population, consistent with findings from diverse clinical and community settings (28, 29). However, a crucial finding of this study is the significant nonlinear “threshold effect” in the relationship between GNRI and the risk of disability. Restricted cubic spline analyses revealed that GNRI exhibited significant nonlinear, inverse associations with the risk of BADL, IADL, and ADL disability (P for nonlinear < 0.05 for all), with risk increasing more sharply below specific GNRI thresholds. This phenomenon may be related to a “metabolic crisis” triggered by severe malnutrition. When GNRI falls below this threshold, it typically signifies severe protein-energy malnutrition, at which point the balance between skeletal muscle protein synthesis and breakdown in the body is completely disrupted, leading to a precipitous decline in muscle mass and strength (i.e., acute or severe sarcopenia). This directly undermines the physiological foundation necessary for maintaining basic activities such as walking, bathing, and dressing (17, 30). This nonlinear relationship underscores that the prevention of BADL disability hinges on early identification and maintaining GNRI above the critical threshold, thereby avoiding a vicious cycle of rapid functional decompensation.

Compared to BADL, the association pattern between GNRI and IADL disability, while also negative, likely involves more complex underlying mechanisms. IADL involves complex activities such as managing finances, shopping, and cooking, which require higher cognitive function, executive ability, and planning. Malnutrition may indirectly impair cognitive function by affecting cerebral nutrient supply, exacerbating neuroinflammation, or concomitant vitamin deficiencies (e.g., B12), thereby impacting IADL performance (31, 32). Our finding that GNRI is associated with IADL disability suggests that nutritional risk is an early signal of complex functional decline.

### 4.2 The mediating role of ABSI

An important finding of this study is the identification of ABSI as a statistically significant mediator of the GNRI-disability associations. This suggests that the relationship between nutritional risk and functional disability may be partially explained by body composition changes reflected in ABSI. However, as mediation analysis in cross-sectional data identifies statistical associations rather than causal pathways, these findings should be interpreted as hypothesis-generating and require confirmation in prospective studies.

For BADL disability, the mediating mechanism of ABSI likely revolves primarily around skeletal muscle health. Central obesity (high ABSI) is a significant driver of intramuscular fat infiltration (i.e., fat deposition within muscles) (33). This ectopic fat deposition not only directly replaces contractile muscle tissue, weakening muscle strength, but the pro-inflammatory adipokines (e.g., TNF-*α*, IL-6) secreted by this fat also create a state of chronic low-grade inflammation both locally and systemically (11, 34). This “inflamm-ageing” environment exacerbates muscle protein breakdown and interferes with insulin signaling, collectively leading to a decline in muscle mass and function (11, 35). Therefore, malnutrition may first lead to the centralization of body fat to the abdomen (increased ABSI), which then damages muscles through the aforementioned mechanisms, ultimately manifesting as BADL difficulties. The strong association of ABSI with BADL disability (highest OR) and its largest mediating proportion in this study support this pathological chain centered on the somatic muscular system.

For IADL disability, the mediating pathway of ABSI may place greater emphasis on effects on cognition and the nervous system. Central obesity is associated with higher cerebrovascular risk, burden of cerebral small vessel disease, and systemic inflammation levels, all of which can impair cognitive function and executive abilities (34, 36). Inflammatory factors and lipotoxic substances released by visceral fat may cross the blood–brain barrier, triggering neuroinflammation and affecting brain regions involved in planning and judgment (37). Furthermore, insulin resistance, which often coexists with central obesity, may directly impact brain energy metabolism and neuronal health (37). Consequently, high ABSI resulting from malnutrition may mediate the association between GNRI and IADL disability by exacerbating cerebrovascular pathology and neuroinflammation, thereby weakening an individual’s ability to handle complex tasks. This explains why individuals with malnutrition accompanied by central obesity may still face instrumental activity disorders even if their physical muscle strength is relatively preserved.

### 4.3 Effect modification by Anemia

This study further found that anemia status significantly modified the strength of the association between GNRI and BADL, IADL, and ADL disability (P for interaction < 0.001 for all), with the association being particularly strong in anemic patients. Anemia and malnutrition often coexist, and our finding of significant effect modification suggests that anemic status may potentiate the association between nutritional risk and functional disability. While this is consistent with the hypothesis of synergistic effects, the cross-sectional design precludes conclusions about temporal ordering or causal direction (38, 39). Reduced tissue oxygen delivery caused by anemia can amplify the muscle metabolic dysfunction and fatigue induced by malnutrition (40). When the nutritional risk indicated by GNRI coexists with anemia, it signifies a dual assault on the physiological reserve of the older adults, potentially significantly lowering the “threshold” for progressing to functional disability and sharply increasing the risk (41, 42). Therefore, in clinical assessment, for older adults with anemia, even if their GNRI indicates only mild risk, they should be considered a very high-risk group for ADL disability, warranting the initiation of more proactive multimodal interventions.

### 4.4 Limitations

Several limitations should be considered when interpreting our findings:

#### 4.4.1 Study design and causality

The cross-sectional design precludes establishing temporal relationships or causal inferences. While mediation analysis identified statistical associations consistent with hypothesized pathways, reverse causality cannot be excluded—for example, ADL disability could lead to reduced physical activity, promoting central obesity and worsening nutritional status. Longitudinal studies with repeated measurements are needed to confirm the directionality of observed associations.

#### 4.4.2 Generalizability

This study was conducted in a single rural county (Donglan) in Guangxi, China, which may limit generalizability to: (1) urban populations with different lifestyle and healthcare access patterns; (2) other rural regions in China with varying socioeconomic conditions, dietary practices, and ethnic compositions; (3) older adult populations in high-income countries with different healthcare systems and nutritional transitions. The study population was predominantly of Zhuang ethnicity, and findings may not directly apply to other ethnic groups. However, the biological relationships examined are likely to be broadly relevant, and our findings provide a foundation for comparative studies in diverse settings.

#### 4.4.3 Measurement and instrument considerations

Although we used validated Chinese-adapted scales for ADL assessment, several measurement considerations warrant mention: (1) Self-reported or proxy-reported functional status may be subject to recall bias or social desirability bias, despite interviewer administration; (2) The PSMS and IADL scales, while widely used, may not capture all dimensions of functional ability relevant to rural Chinese older adults (e.g., agricultural work, traditional activities); (3) GNRI, while a validated nutritional screening tool, does not capture all aspects of nutritional status (e.g., micronutrient deficiencies, dietary diversity); (4) Single-timepoint biomarker measurements may not reflect chronic inflammatory or metabolic status and are subject to day-to-day variability; (5) Composite biomarkers (SIRI, AISI, METS-IR, TyG), while reflecting integrated physiological processes, have not been extensively validated in older rural Chinese populations.

#### 4.4.4 Residual confounding

Despite comprehensive adjustment for multiple covariates, residual confounding by unmeasured factors remains possible. These may include: dietary quality and patterns, physical activity levels, social support networks, depression and psychological factors, medication use (particularly polypharmacy), and genetic predisposition. Future studies incorporating these variables may provide additional insights.

#### 4.4.5 Data timeliness

The data for this study were collected between 2016 and 2018, and we acknowledge the potential limitations associated with this time lag. However, several factors support the continued relevance of our findings. First, the physiological and biological relationships examined—such as the association between nutritional status, body composition, and functional disability—are grounded in fundamental gerontological and metabolic processes that are unlikely to change substantially over relatively short time periods. Second, the demographic and socioeconomic characteristics of rural aging populations in China, particularly in less-developed regions like Guangxi, have evolved gradually, with persistent challenges related to healthcare access, nutritional transitions, and functional decline (43). Third, recent large-scale studies continue to report similar prevalence rates of malnutrition (8–12%) and ADL disability (25–35%) among rural Chinese older adults, suggesting that the underlying phenomena remain relevant (44, 45). Nevertheless, we acknowledge that more recent data could capture the impact of contemporary healthcare policies, economic development, and nutritional transitions in rural China. Future studies with updated data collection are warranted to confirm and extend our findings in the current context.

#### 4.4.6 Selection bias

Although we achieved a high participation rate, non-participants may have differed from participants in ways that could affect generalizability. Excluded individuals with missing data (*n* = 452, 9.3%) were more likely to be older and have lower education, potentially introducing selection bias.

## 5 Conclusion

This study demonstrates that poorer nutritional status, as indicated by a lower Geriatric Nutritional Risk Index (GNRI), is independently associated with an increased risk of both basic and instrumental activities of daily living (BADL and IADL) disability among older adults in rural China. Central obesity, characterized by a higher A Body Shape Index (ABSI), serves as a significant mediator in these associations. The relationships between GNRI and BADL, IADL, and ADL disability are nonlinear, and the observed associations are notably stronger among individuals with anemia.

These findings have important implications for both clinical practice and public health policy. At the clinical level, the results support the use of GNRI as a routine screening tool to identify older adults at risk of functional decline and underscore the importance of integrating body composition optimization—particularly the prevention of central obesity—into nutritional intervention strategies, especially for anemic patients. From a public health perspective, implementing combined screening for nutritional risk and central obesity assessment in rural primary care settings may offer a more effective approach to delaying functional disability than either strategy alone. Future policy initiatives should consider strengthening community-based nutritional support systems and promoting physical activity environments conducive to maintaining healthy body composition.

Longitudinal studies and intervention trials are warranted to confirm the causal pathways identified and to evaluate the effectiveness of integrated nutritional and body composition management interventions in preventing or delaying ADL disability, thereby informing evidence-based guidelines for this vulnerable population.

## Data Availability

The original contributions presented in the study are included in the article/Supplementary material, further inquiries can be directed to the corresponding author.

## Footnotes

1
https://www.medsta.cn
