# Supplementary material for: A Body Shape Index mediates the associations of geriatric nutritional risk index with activities of daily living disability in an older rural population in Guangxi, China
Source: Front Public Health. 2026 Apr 24;14:1808377. doi: 10.3389/fpubh.2026.1808377 (PMC13152727; doi:10.3389/fpubh.2026.1808377)

**Supplementary Material**

**A Body Shape Index Mediates the Associations of Geriatric Nutritional Risk Index with Activities of Daily Living Disability in an Older Rural Population in Guangxi, China**

Wenjie Liang^1#^, Haiyan Lu^2#^, Kaiyong Huang^2^, Li Yang^2*^

**Table S1** the groups and definitions of covariates

**Table S2** the total missing data of this study

**Table S3** Multicollinearity between independence and covariate

**Table S4** Mediation effects of SIRI, AISI, METS–IR, ABSI, and TyG in the association of GNRI with BADL, IADL, and ADL disability

**Table S5** Mediation effects of SIRI, AISI, METS–IR, ABSI, and TyG in the association of GNRI with BADL, IADL, and ADL disability stratified by sex

**Table S6** Mediation effects of SIRI, AISI, METS–IR, ABSI, and TyG in the association of GNRI with BADL, IADL, and ADL disability stratified by age

**Table S7** Mediation effects of SIRI, AISI, METS–IR, ABSI, and TyG in the association of GNRI with BADL, IADL, and ADL disability stratified by hypertension

**Table S8** Mediation effects of SIRI, AISI, METS–IR, ABSI, and TyG in the association of GNRI with BADL, IADL, and ADL disability stratified by anemia

**Table S9** Association of GNRI (categorized by quartiles) with BADL, IADL, and ADL disability

**Table S10** Association of GNRI (categorized by quartiles) with SIRI, AISI, METS–IR, ABSI, and TyG

**Table S11** Association of GNRI with BADL, IADL, and ADL disability by modified Poisson regression analysis

**Table S12** Association of SIRI, ABSI, METS–IR, ABSI, and TyG with BADL, IADL, and ADL disability by modified Poisson regression analysis

**Table S13** Association of GNRI with BADL, IADL, and ADL disability in population without metabolic syndrome

**Table S14** Mediation effects of SIRI, AISI, METS–IR, ABSI, and TyG in the association of GNRI with BADL, IADL, and ADL disability stratified in population without metabolic syndrome

Figure S1 The non–linear relationships of SIRI, AISI, METS–IR, ABSI, and TyG with BADL, IADL, and ADL disability.

Figure S2 The non–linear relationships of mediators with GNRI. (A) SIRI, (B) AISI, (C) METS–IR, (D) ABSI, (E) TyG.

Figure S3 The non–linear relationships of GNRI with BADL, IADL, and ADL disability in population without metabolic syndrome.

Figure S4 The non–linear relationships of mediators with BADL, IADL, and ADL disability in population without metabolic syndrome.

Table S1 The groups and definitions of covariates

| Covariates | Groups | Definition |
| --- | --- | --- |
| **Demographics variables** | | |
| Age | 60–69 years | – |
|  | ≥70 years | – |
| Gender | male | – |
|  | female | – |
| Marital status | single | including divorced, widowed, single |
|  | partnered | married and partnered |
| Educational attainment | less than primary school | – |
|  | primary school and above | – |
| Annual income | ＜10000 renminbi | – |
|  | ≥10000 renminbi | – |
| **Lifestyle factors** |  | Participants who had always smoked ≥1 cigarette per day over the past 6 months were defined as current smokers |
| Smoking status | no | ex–smokers and nonsmokers (participants who had quit smoking at the time of the interview were defined as ex–smokers;  those who never smoked in lifetime were defined as nonsmokers) |
|  | yes | current smokers (participants who had always smoked ≥1 cigarette per day over the past 6 months were defined as current smokers) |
| Alcohol consumption | no | nondrinkers and ex–drinkers (participants who had quit drinking at the time of the survey were defined as ex–drinkers;  those who never drank alcohol in their lifetime were defined as nondrinkers) |
|  | yes | current drinkers (participants who had always smoked ≥1 cigarette per day over the past 6 months were defined as current smokers) |
| **Chronic diseases** |  | the history of chronic diseases was obtained by asking participants if |
| Cerebrovascular disease | no | participants who had not been diagnosed by a doctor with cerebrovascular disease |
|  | yes | participants who had been diagnosed by a doctor with cerebrovascular disease |
| Rheumatism | no | participants who had not been diagnosed by a doctor with rheumatism |
|  | yes | participants who had been diagnosed by a doctor with rheumatism |
| Osteoarthropathy | no | participants who had not been diagnosed by a doctor with osteoarthropathy |
|  | yes | participants who had been diagnosed by a doctor with osteoarthropathy |
| Hypertension | no | participants who had not been diagnosed by a doctor with hypertension |
|  | yes | participants who had been diagnosed by a doctor with hypertension |
| Diabetes | no | participants who had not been diagnosed by a doctor with diabetes |
|  | yes | participants who had been diagnosed by a doctor with diabetes |
| **Physical examination indicators** | | |
| Anemia | no | male: hemoglobin＜120 g/L, female: hemoglobin＜110 g/L |
|  | yes | male: hemoglobin≥120 g/L, female: hemoglobin≥110 g/L |
| eGFR | normal | ≥ 60 mL/min/1.73 m² |
|  | low | ＜60 mL/min/1.73 m² |
| Aspartate aminotransferase | normal | ≤ 35U/L |
|  | high | ＞35U/L |
| Alanine aminotransferase | normal | ≤ 40U/L |
|  | high | ＞40U/L |

Table S2 the total missing data of this study

| Variables | Total n(%) | GNRI n(%) | |
| --- | --- | --- | --- |
|  |  | Normal | Low |
| Sex | 0 (0.00) | 0 (0.00) | 0 (0.00) |
| Age | 0 (0.00) | 0 (0.00) | 0 (0.00) |
| Marital status | 7 (0.16) | 6 (0.14) | 1 (0.02) |
| Educational attainment | 10 (0.23) | 9 (0.20) | 1 (0.02) |
| Annual income | 42 (0.95) | 35 (0.80) | 7 (0.16) |
| Cerebrovascular disease | 0 (0.00) | 0 (0.00) | 0 (0.00) |
| Osteoarthropathy | 1 (0.02) | 1 (0.02) | 0 (0.00) |
| Rheumatism | 3 (0.07) | 3 (0.07) | 0 (0.00) |
| Hypertension | 2 (0.05) | 1 (0.02) | 1 (0.02) |
| Diabetes | 0 (0.00) | 0 (0.00) | 0 (0.00) |
| eGFR | 41 (0.93) | 36 (0.82) | 5 (0.11) |
| Anemia | 13 (0.30) | 10 (0.23) | 3 (0.07) |
| ALT | 0 (0.00) | 0 (0.00) | 0 (0.00) |
| AST | 0 (0.00) | 0 (0.00) | 0 (0.00) |
| Smoking status | 5 (0.11) | 3 (0.07) | 2 (0.05) |
| Alcohol consumption | 23 (0.52) | 22 (0.50) | 1 (0.02) |
| GNRI | 0 (0.00) | – | – |
| BADL | 0 (0.00) | 0 (0.00) | 0 (0.00) |
| IADL | 0 (0.00) | 0 (0.00) | 0 (0.00) |
| ADL | 0 (0.00) | 0 (0.00) | 0 (0.00) |
| SIRI | 11 (0.25) | 8 (0.18) | 3 (0.07) |
| AISI | 13 (0.30) | 10 (0.23) | 3 (0.07) |
| METSIR | 3 (0.07) | 2 (0.05) | 1 (0.02) |
| ABSI | 0 (0.00) | 0 (0.00) | 0 (0.00) |
| TyG | 2 (0.05) | 1 (0.02) | 1 (0.02) |

GNRI, geriatric nutritional risk index; eGFR, estimated glomerular filtration rate; AST, aspartate aminotransferase; ALT, alanine aminotransferase; SIRI, systemic inflammation response index; AISI, aggregate index of systemic inflammation; METS–IR, metabolic score for insulin resistance; ABSI, A body shape index; TyG, triglyceride–glucose index; BADL, basic activities of daily living; IADL, instrumental activities of daily living; ADL, activities of daily living.

Table S3 Multicollinearity between independence and covariate

| Variables | Variance inflation factor | Tolerance |
| --- | --- | --- |
| GNRI | 1.07 | 0.94 |
| Sex | 2.37 | 0.42 |
| Age | 1.17 | 0.85 |
| Marital status | 1.16 | 0.86 |
| Educational attainment | 1.49 | 0.67 |
| Annual income | 1.02 | 0.98 |
| Cerebrovascular disease | 1.01 | 0.99 |
| Osteoarthropathy | 1.03 | 0.97 |
| Rheumatism | 1.03 | 0.97 |
| Hypertension | 1.06 | 0.94 |
| Diabetes | 1.10 | 0.91 |
| eGFR | 1.11 | 0.90 |
| Anemia | 1.07 | 0.93 |
| ALT | 1.19 | 0.84 |
| AST | 1.22 | 0.82 |
| Alcohol consumption | 1.59 | 0.63 |
| Smoking status | 1.39 | 0.72 |
| SIRI | 2.27 | 0.44 |
| AISI | 2.26 | 0.44 |
| METS–IR | 1.30 | 0.77 |
| ABSI | 1.07 | 0.94 |
| TyG | 1.39 | 0.72 |

GNRI, geriatric nutritional risk index; eGFR, estimated glomerular filtration rate; AST, aspartate aminotransferase; ALT, alanine aminotransferase; SIRI, systemic inflammation response index; AISI, aggregate index of systemic inflammation; METS–IR, metabolic score for insulin resistance; ABSI, A body shape index; TyG, triglyceride–glucose index.

Table S4 Mediation effects of SIRI, AISI, METS–IR, ABSI, and TyG in the association of GNRI with BADL, IADL, and ADL disability

| Mediators | ADE | |  | ACME | | PME (%) |
| --- | --- | --- | --- | --- | --- | --- |
|  | β (95%CI) | *P* value |  | β (95%CI) | *P* value |  |
| BADL disability |  |  |  |  |  |  |
| SIRI | 0.067 (0.040, 0.099) | < 0.001 |  | 0.001 (0.000, 0.002) | 0.14 | 1.05 |
| AISI | 0.067 (0.038, 0.098) | < 0.001 |  | 0.001 (0.000, 0.002) | 0.16 | 1.05 |
| METSIR | 0.068 (0.043, 0.099) | < 0.001 |  | -0.002 (-0.005, 0.000) | **0.02** | **-3.57** |
| ABSI | 0.043 (0.011, 0.074) | 0.02 |  | 0.019 (0.013, 0.026) | **< 0.001** | **30.41** |
| TyG | 0.069 (0.044, 0.099) | < 0.001 |  | -0.003 (-0.006, 0.000) | 0.06 | -4.04 |
| IADL disability |  |  |  |  |  |  |
| SIRI | 0.103 (0.065, 0.151) | < 0.001 |  | 0.000 (-0.002, 0.001) | 0.80 | -0.20 |
| AISI | 0.103 (0.066, 0.145) | < 0.001 |  | -0.001 (-0.002, 0.001) | 0.58 | -0.52 |
| METSIR | 0.098 (0.058, 0.144) | < 0.001 |  | 0.003 (0.000, 0.007) | **0.02** | **3.37** |
| ABSI | 0.077 (0.030, 0.136) | < 0.001 |  | 0.025 (0.016, 0.036) | **< 0.001** | **24.39** |
| TyG | 0.101 (0.065, 0.148) | < 0.001 |  | 0.001 (-0.003, 0.004) | 0.90 | 0.55 |
| ADL disability |  |  |  |  |  |  |
| SIRI | 0.065 (0.039, 0.093) | < 0.001 |  | 0.001 (0.000, 0.002) | 0.12 | 1.06 |
| AISI | 0.065 (0.037, 0.093) | < 0.001 |  | 0.001 (0.000, 0.002) | 0.12 | 1.09 |
| METSIR | 0.066 (0.034, 0.098) | < 0.001 |  | -0.002 (-0.005, 0.000) | **0.02** | **-3.28** |
| ABSI | 0.040 (0.014, 0.081) | < 0.001 |  | 0.018 (0.012, 0.025) | **< 0.001** | **31.07** |
| TyG | 0.066 (0.034, 0.097) | < 0.001 |  | -0.002 (-0.005, 0.000) | 0.06 | -3.21 |

SIRI, systemic inflammation response index; AISI, aggregate index of systemic inflammation; METS–IR, metabolic score for insulin resistance; ABSI, A body shape index; TyG, triglyceride–glucose index; GNRI, geriatric nutritional risk index; ADE, average direct effects; ACME, average causal mediation effects (indirect effect); PME, proportion of mediation effect; BADL, basic activities of daily living; IADL, instrumental activities of daily living; ADL, activities of daily living.

Table S5 Mediation effects of SIRI, AISI, METS–IR, ABSI, and TyG in the association of GNRI with BADL, IADL, and ADL disability stratified by sex

| Mediators | Sex | ADE |  |  | ACME |  | PME (%) |
| --- | --- | --- | --- | --- | --- | --- | --- |
|  |  | β (95%CI) | *P* value |  | β (95%CI) | *P* value |  |
| BADL disability |  |  |  |  |  |  |  |
| SIRI | Male | 0.069 (0.033, 0.110) | <.001 |  | 0.001 (-0.001, 0.002) | 0.32 | 1.06 |
|  | Female | 0.056 (0.015, 0.106) | <.001 |  | 0.001 (-0.002, 0.003) | 0.58 | 1.34 |
| AISI | Male | 0.069 (0.037, 0.105) | <.001 |  | 0.001 (-0.001, 0.003) | 0.46 | 1.21 |
|  | Female | 0.056 (0.012, 0.104) | <.001 |  | 0.001 (-0.001, 0.003) | 0.48 | 1.24 |
| METS–IR | Male | 0.072 (0.037, 0.119) | <.001 |  | -0.005 (-0.009, 0.000) | 0.1 | -7.06 |
|  | Female | 0.058 (0.018, 0.107) | <.001 |  | 0.000 (-0.002, 0.004) | 0.72 | 0.58 |
| ABSI | Male | 0.042 (0.007, 0.078) | 0.02 |  | 0.021 (0.014, 0.031) | **<.001** | **32.96** |
|  | Female | 0.039 (0.003, 0.091) | 0.04 |  | 0.013 (0.005, 0.026) | **<.001** | **25.58** |
| TyG | Male | 0.072 (0.034, 0.109) | <.001 |  | -0.004 (-0.009, 0.001) | 0.18 | -5.20 |
|  | Female | 0.060 (0.022, 0.107) | <.001 |  | -0.002 (-0.005, 0.001) | 0.18 | -2.67 |
| IADL disability |  |  |  |  |  |  |  |
| SIRI | Male | 0.137 (0.077, 0.206) | <.001 |  | 0.000 (-0.003, 0.002) | 0.86 | -0.22 |
|  | Female | 0.047 (-0.010 ~ 0.114) | 0.12 |  | -0.000 (-0.002 ~ 0.002) | 0.86 | -0.20 |
| AISI | Male | 0.136 (0.081, 0.189) | <.001 |  | 0.000 (-0.002, 0.002) | 0.72 | -0.19 |
|  | Female | 0.048 (-0.005 ~ 0.109) | 0.08 |  | -0.001 (-0.009 ~ 0.002) | 0.5 | -2.44 |
| METS–IR | Male | 0.128 (0.062, 0.172) | <.001 |  | 0.007 (0.000, 0.014) | 0.1 | 4.85 |
|  | Female | 0.047 (-0.011 ~ 0.113) | 0.12 |  | -0.000 (-0.003 ~ 0.001) | 0.86 | -0.82 |
| ABSI | Male | 0.109 (0.044, 0.165) | <.001 |  | 0.028 (0.016, 0.042) | **<.001** | **20.60** |
|  | Female | 0.027 (-0.025 ~ 0.092) | 0.32 |  | 0.017 (0.006 ~ 0.032) | **<.001** | **38.72** |
| TyG | Male | 0.135 (0.081, 0.205) | <.001 |  | -0.001 (-0.008, 0.006) | 0.98 | -0.52 |
|  | Female | 0.046 (-0.013 ~ 0.114) | 0.12 |  | 0.000 (-0.003 ~ 0.003) | 0.68 | 1.06 |
| ADL disability |  |  |  |  |  |  |  |
| SIRI | Male | 0.073 (0.035, 0.113) | <.001 |  | 0.001 (0.000, 0.003) | 0.24 | 0.97 |
|  | Female | 0.047 (0.012, 0.088) | <.001 |  | 0.001 (-0.002, 0.003) | 0.58 | 1.51 |
| AISI | Male | 0.073 (0.035, 0.110) | <.001 |  | 0.001 (-0.001, 0.003) | 0.3 | 1.09 |
|  | Female | 0.047 (0.010, 0.098) | 0.02 |  | 0.001 (-0.001, 0.003) | 0.42 | 1.51 |
| METS–IR | Male | 0.075 (0.043, 0.119) | <.001 |  | -0.004 (-0.008, 0.001) | 0.2 | -5.13 |
|  | Female | 0.049 (0.014, 0.091) | <.001 |  | 0.000 (-0.002, 0.004) | 0.76 | 0.72 |
| ABSI | Male | 0.046 (0.015, 0.082) | 0.02 |  | 0.021 (0.013, 0.032) | **<.001** | **31.20** |
|  | Female | 0.030 (-0.006, 0.073) | 0.12 |  | 0.013 (0.005, 0.025) | **<.001** | **29.74** |
| TyG | Male | 0.075 (0.037, 0.118) | <.001 |  | -0.003 (-0.008, 0.001) | 0.22 | -4.57 |
|  | Female | 0.050 (0.018, 0.092) | <.001 |  | -0.001 (-0.004, 0.001) | 0.4 | -1.89 |

SIRI, systemic inflammation response index; AISI, aggregate index of systemic inflammation; METS–IR, metabolic score for insulin resistance; ABSI, A body shape index; TyG, triglyceride–glucose index; GNRI, geriatric nutritional risk index; ADE, average direct effects; ACME, average causal mediation effects (indirect effect); PME, proportion of mediation effect; BADL, basic activities of daily living; IADL, instrumental activities of daily living; ADL, activities of daily living.

Table S6 Mediation effects of SIRI, AISI, METS–IR, ABSI, and TyG in the association of GNRI with BADL, IADL, and ADL disability stratified by age

| Mediators | Age group  (years) | ADE |  |  | ACME |  | PME(%) |
| --- | --- | --- | --- | --- | --- | --- | --- |
|  |  | β (95%CI) | *P* value |  | β (95%CI) | *P* value |  |
| BADL disability |  |  |  |  |  |  |  |
| SIRI | 60-69 | 0.000 (-0.001, 0.001) | **0.02** |  | 0.048 (0.006, 0.093) | 0.84 | -0.26 |
|  | ≥ 70 | 0.002 (-0.001, 0.005) | <.001 |  | 0.087 (0.042, 0.147) | 0.16 | 1.82 |
| AISI | 60-69 | 0.000 (-0.001, 0.002) | **0.02** |  | 0.047 (0.004, 0.098) | 0.82 | 0.14 |
|  | ≥ 70 | 0.002 (-0.001, 0.006) | <.001 |  | 0.087 (0.043, 0.147) | 0.16 | 1.99 |
| METS–IR | 60-69 | 0.000 (-0.003, 0.004) | 0.04 |  | 0.049 (0.013, 0.109) | 0.86 | -0.07 |
|  | ≥ 70 | -0.003 (-0.009, 0.000) | <.001 |  | 0.090 (0.054, 0.137) | 0.08 | -3.97 |
| ABSI | 60-69 | 0.005 (0.001, 0.014) | 0.06 |  | 0.041 (0.000, 0.087) | **0.04** | **11.90** |
|  | ≥ 70 | 0.036 (0.024, 0.053) | 0.08 |  | 0.046 (-0.003, 0.100) | **<.001** | **44.03** |
| TyG | 60-69 | 0.000 (-0.004, 0.003) | 0.02 |  | 0.049 (0.004, 0.097) | 0.88 | -0.89 |
|  | ≥ 70 | -0.006 (-0.013, -0.002) | <.001 |  | 0.093 (0.056, 0.138) | **0.02** | **-7.17** |
| IADL disability |  |  |  |  |  |  |  |
| SIRI | 60-69 | 0.134 (0.062, 0.198) | <.001 |  | 0.000 (-0.003, 0.002) | 0.90 | -0.16 |
|  | ≥ 70 | 0.088 (0.038, 0.147) | <.001 |  | -0.002 (-0.007, 0.001) | 0.30 | -2.12 |
| AISI | 60-69 | 0.133 (0.070, 0.189) | <.001 |  | 0.000 (-0.002, 0.003) | 0.96 | 0.08 |
|  | ≥ 70 | 0.089 (0.038, 0.148) | <.001 |  | -0.002 (-0.008, 0.001) | 0.20 | -2.70 |
| METS–IR | 60-69 | 0.126 (0.052, 0.195) | <.001 |  | 0.007 (0.001, 0.012) | 0.02 | 4.95 |
|  | ≥ 70 | 0.083 (0.019, 0.144) | <.001 |  | 0.002 (-0.001, 0.007) | 0.32 | 1.95 |
| ABSI | 60-69 | 0.124 (0.033, 0.181) | 0.02 |  | 0.009 (0.002, 0.018) | **0.04** | **6.85** |
|  | ≥ 70 | 0.044 (-0.002, 0.097) | 0.08 |  | 0.043 (0.022, 0.062) | **<.001** | **49.39** |
| TyG | 60-69 | 0.133 (0.044, 0.192) | <.001 |  | 0.001 (-0.003, 0.006) | 0.62 | 0.59 |
|  | ≥ 70 | 0.085 (0.019, 0.151) | <.001 |  | -0.001 (-0.006, 0.005) | 0.86 | -0.69 |
| ADL disability |  |  |  |  |  |  |  |
| SIRI | 60-69 | 0.038 (-0.001, 0.103) | 0.06 |  | 0.000 (-0.002, 0.001) | 0.72 | -0.36 |
|  | ≥ 70 | 0.092 (0.049, 0.142) | <.001 |  | 0.001 (0.000, 0.004) | 0.14 | 1.59 |
| AISI | 60-69 | 0.038 (0.004, 0.094) | 0.04 |  | 0.000 (-0.001, 0.002) | 0.88 | 0.19 |
|  | ≥ 70 | 0.091 (0.049, 0.141) | <.001 |  | 0.002 (-0.001, 0.005) | 0.22 | 1.90 |
| METS–IR | 60-69 | 0.039 (0.000, 0.086) | 0.06 |  | 0.001 (-0.001, 0.004) | 0.34 | 2.93 |
|  | ≥ 70 | 0.094 (0.050, 0.139) | <.001 |  | -0.004 (-0.010, 0.000) | **0.02** | **-3.97** |
| ABSI | 60-69 | 0.033 (-0.004, 0.098) | 0.16 |  | 0.004 (-0.001, 0.011) | 0.08 | 11.00 |
|  | ≥ 70 | 0.048 (0.003, 0.097) | 0.06 |  | 0.037 (0.023, 0.050) | **<.001** | **43.83** |
| TyG | 60-69 | 0.038 (-0.001, 0.106) | 0.08 |  | 0.001 (-0.002, 0.003) | 0.74 | 1.71 |
|  | ≥ 70 | 0.097 (0.052, 0.143) | <.001 |  | -0.006 (-0.012, -0.001) | **0.02** | **-6.76** |

SIRI, systemic inflammation response index; AISI, aggregate index of systemic inflammation; METS–IR, metabolic score for insulin resistance; ABSI, A body shape index; TyG, triglyceride–glucose index; GNRI, geriatric nutritional risk index; ADE, average direct effects; ACME, average causal mediation effects (indirect effect); PME, proportion of mediation effect; BADL, basic activities of daily living; IADL, instrumental activities of daily living; ADL, activities of daily living.

Table S7 Mediation effects of SIRI, AISI, METS–IR, ABSI, and TyG in the association of GNRI with BADL, IADL, and ADL disability stratified by hypertension

| Mediators | Hypertension | ADE |  |  | ACME |  | PME (%) |
| --- | --- | --- | --- | --- | --- | --- | --- |
|  |  | β (95%CI) | *P* value |  | β (95%CI) | *P* value |  |
| BADL disability |  |  |  |  |  |  |  |
| SIRI | No | 0.076 (0.038, 0.118) | <.001 |  | 0.001 (-0.001, 0.003) | 0.40 | 0.85 |
|  | Yes | 0.048 (0.004, 0.095) | 0.04 |  | 0.001 (-0.001, 0.004) | 0.52 | 1.18 |
| AISI | No | 0.076 (0.033, 0.110) | <.001 |  | 0.001 (-0.001, 0.005) | 0.36 | 0.97 |
|  | Yes | 0.048 (0.004, 0.095) | 0.04 |  | 0.001 (-0.001, 0.002) | 0.52 | 1.14 |
| METS–IR | No | 0.077 (0.039, 0.121) | <.001 |  | -0.003 (-0.007, -0.001) | 0.02 | -4.64 |
|  | Yes | 0.050 (0.004, 0.101) | 0.04 |  | -0.001 (-0.005, 0.001) | 0.42 | -2.81 |
| ABSI | No | 0.053 (0.015, 0.088) | <.001 |  | 0.017 (0.008, 0.028) | **<.001** | **24.06** |
|  | Yes | 0.025 (-0.020, 0.070) | 0.28 |  | 0.021 (0.011, 0.032) | **<.001** | **45.43** |
| TyG | No | 0.079 (0.038, 0.113) | <.001 |  | -0.003 (-0.008, 0.000) | 0.06 | -4.35 |
|  | Yes | 0.051 (0.007, 0.100) | 0.04 |  | -0.002 (-0.006, 0.001) | 0.16 | -4.69 |
| IADL disability |  |  |  |  |  |  |  |
| SIRI | No | 0.112 (0.051, 0.172) | <.001 |  | 0.000 (-0.001, 0.003) | 0.68 | 0.44 |
|  | Yes | 0.079 (0.018, 0.138) | <.001 |  | -0.001 (-0.003, 0.001) | 0.66 | -0.88 |
| AISI | No | 0.112 (0.056, 0.165) | <.001 |  | 0.000 (-0.001, 0.005) | 0.76 | 0.24 |
|  | Yes | 0.080 (0.019, 0.138) | <.001 |  | -0.002 (-0.006, 0.001) | 0.32 | -2.10 |
| METS–IR | No | 0.110 (0.055, 0.156) | <.001 |  | 0.002 (-0.003, 0.007) | 0.52 | 1.40 |
|  | Yes | 0.073 (0.017, 0.134) | <.001 |  | 0.004 (0.000, 0.011) | 0.12 | 5.27 |
| ABSI | No | 0.089 (0.032, 0.148) | <.001 |  | 0.023 (0.012, 0.039) | **<.001** | **20.81** |
|  | Yes | 0.052 (-0.002, 0.109) | 0.08 |  | 0.027 (0.011, 0.042) | **<.001** | **33.79** |
| TyG | No | 0.113 (0.062, 0.170) | <.001 |  | -0.001 (-0.006, 0.004) | 0.46 | -1.29 |
|  | Yes | 0.077 (0.015, 0.137) | <.001 |  | 0.002 (-0.002, 0.006) | 0.60 | 1.93 |
| ADL disability |  |  |  |  |  |  |  |
| SIRI | No | 0.068 (0.036, 0.111) | <.001 |  | 0.001 (0.000, 0.003) | 0.48 | 0.87 |
|  | Yes | 0.053 (0.010, 0.099) | 0.02 |  | 0.001 (-0.001, 0.004) | 0.54 | 1.10 |
| AISI | No | 0.067 (0.027, 0.107) | <.001 |  | 0.001 (-0.001, 0.004) | 0.40 | 0.97 |
|  | Yes | 0.053 (0.011, 0.099) | 0.02 |  | 0.001 (-0.001, 0.003) | 0.46 | 1.16 |
| METS–IR | No | 0.068 (0.035, 0.110) | <.001 |  | -0.003 (-0.006, 0.000) | 0.08 | -4.27 |
|  | Yes | 0.055 (0.011, 0.103) | 0.02 |  | -0.001 (-0.004, 0.001) | 0.38 | -2.36 |
| ABSI | No | 0.043 (0.011, 0.080) | 0.02 |  | 0.016 (0.008, 0.027) | **<.001** | **27.04** |
|  | Yes | 0.031 (-0.014, 0.074) | 0.2 |  | 0.021 (0.012, 0.032) | **<.001** | **40.21** |
| TyG | No | 0.069 (0.028, 0.100) | <.001 |  | -0.002 (-0.006, 0.001) | 0.16 | -3.17 |
|  | Yes | 0.056 (0.014, 0.102) | <.001 |  | -0.002 (-0.005, 0.001) | 0.22 | -3.71 |

SIRI, systemic inflammation response index; AISI, aggregate index of systemic inflammation; METS–IR, metabolic score for insulin resistance; ABSI, A body shape index; TyG, triglyceride–glucose index; GNRI, geriatric nutritional risk index; ADE, average direct effects; ACME, average causal mediation effects (indirect effect); PME, proportion of mediation effect; BADL, basic activities of daily living; IADL, instrumental activities of daily living; ADL, activities of daily living.

Table S8 Mediation effects of SIRI, AISI, METS–IR, ABSI, and TyG in the association of GNRI with BADL, IADL, and ADL disability stratified by anemia

| Mediators | Anemia | ADE |  |  | ACME |  | PME (%) |
| --- | --- | --- | --- | --- | --- | --- | --- |
|  |  | β (95%CI) | *P* value |  | β (95%CI) | *P* value |  |
| BADL disability |  |  |  |  |  |  |  |
| SIRI | No | 0.008 (-0.028, 0.046) | 0.72 |  | 0.001 (-0.001, 0.003) | 0.32 | 10.51 |
|  | Yes | 0.118 (0.069, 0.170) | <.001 |  | 0.000 (-0.001, 0.003) | 0.60 | 0.25 |
| AISI | No | 0.008 (-0.027, 0.047) | 0.72 |  | 0.001 (0.000, 0.004) | 0.12 | 10.41 |
|  | Yes | 0.117 (0.062, 0.179) | <.001 |  | 0.000 (-0.002, 0.003) | 0.50 | 0.38 |
| METS–IR | No | 0.012 (-0.020, 0.048) | 0.58 |  | -0.003 (-0.009, 0.000) | 0.08 | -30.12 |
|  | Yes | 0.117 (0.079, 0.173) | <.001 |  | -0.001 (-0.004, 0.001) | 0.48 | -0.76 |
| ABSI | No | -0.001 (-0.034, 0.038) | 0.98 |  | 0.007 (0.002, 0.013) | **0.02** | **110.41** |
|  | Yes | 0.078 (0.037, 0.122) | <.001 |  | 0.034 (0.018, 0.049) | **<.001** | **30.08** |
| TyG | No | 0.011 (-0.025, 0.052) | 0.64 |  | -0.002 (-0.005, 0.001) | 0.24 | -18.19 |
|  | Yes | 0.119 (0.080, 0.176) | <.001 |  | -0.003 (-0.008, 0.000) | 0.08 | -2.40 |
| IADL disability |  |  |  |  |  |  |  |
| SIRI | No | 0.011 (-0.058, 0.078) | 0.82 |  | 0.000 (-0.002, 0.003) | 0.82 | 3.18 |
|  | Yes | 0.186 (0.137, 0.246) | <.001 |  | -0.001 (-0.003, 0.002) | 0.76 | -0.33 |
| AISI | No | 0.011 (-0.058, 0.078) | 0.84 |  | 0.000 (-0.003, 0.003) | 0.80 | 2.57 |
|  | Yes | 0.185 (0.132, 0.250) | <.001 |  | -0.001 (-0.004, 0.002) | 0.56 | -0.32 |
| METS–IR | No | 0.007 (-0.053, 0.088) | 0.84 |  | 0.004 (-0.003, 0.012) | 0.46 | 33.89 |
|  | Yes | 0.179 (0.119, 0.239) | <.001 |  | 0.002 (-0.002, 0.009) | 0.30 | 1.20 |
| ABSI | No | 0.001 (-0.062, 0.066) | 0.96 |  | 0.009 (0.002, 0.017) | **0.02** | **93.28** |
|  | Yes | 0.139 (0.078, 0.204) | <.001 |  | 0.048 (0.028, 0.073) | **<.001** | **25.57** |
| TyG | No | 0.013 (-0.054, 0.083) | 0.7 |  | -0.002 (-0.009, 0.003) | 0.50 | -15.19 |
|  | Yes | 0.179 (0.121, 0.241) | <.001 |  | 0.003 (-0.001, 0.009) | 0.18 | 1.53 |
| ADL disability |  |  |  |  |  |  |  |
| SIRI | No | 0.009 (-0.022, 0.051) | 0.78 |  | 0.001 (-0.001, 0.004) | 0.24 | 9.83 |
|  | Yes | 0.117 (0.067, 0.164) | <.001 |  | 0.000 (-0.001, 0.003) | 0.68 | 0.19 |
| AISI | No | 0.009 (-0.025, 0.050) | 0.76 |  | 0.001 (0.000, 0.004) | 0.16 | 9.78 |
|  | Yes | 0.117 (0.062, 0.172) | <.001 |  | 0.000 (-0.002, 0.003) | 0.52 | 0.38 |
| METS–IR | No | 0.013 (-0.020, 0.054) | 0.62 |  | -0.002 (-0.006, 0.001) | 0.22 | -19.08 |
|  | Yes | 0.116 (0.081, 0.175) | <.001 |  | -0.001 (-0.004, 0.001) | 0.44 | -0.83 |
| ABSI | No | 0.000 (-0.028, 0.044) | 0.96 |  | 0.006 (0.002, 0.011) | **<.001** | **94.06** |
|  | Yes | 0.077 (0.031, 0.121) | <.001 |  | 0.034 (0.019, 0.049) | **<.001** | **30.44** |
| TyG | No | 0.011 (-0.021, 0.055) | 0.64 |  | -0.001 (-0.005, 0.003) | 0.68 | -6.67 |
|  | Yes | 0.119 (0.084, 0.178) | <.001 |  | -0.003 (-0.007, 0.000) | 0.08 | -2.36 |

SIRI, systemic inflammation response index; AISI, aggregate index of systemic inflammation; METS–IR, metabolic score for insulin resistance; ABSI, A body shape index; TyG, triglyceride–glucose index; GNRI, geriatric nutritional risk index; ADE, average direct effects; ACME, average causal mediation effects (indirect effect); PME, proportion of mediation effect; BADL, basic activities of daily living; IADL, instrumental activities of daily living; ADL, activities of daily living. The mediation effects are estimated based on the fully adjusted model, which adjusted for sex, age, marital status, educational attainment, annual income, cerebrovascular disease, rheumatism, osteoarthropathy, hypertension, diabetes, estimated glomerular filtration rate, anemia, alanine aminotransferase, aspartate aminotransferase, smoking status, and alcohol consumption.

Table S9 Association of GNRI (categorized by quartiles) with BADL, IADL, and ADL disability

| Models | GNRI, OR (95%CI) | | | | P for trend | Per in-quantile increase |
| --- | --- | --- | --- | --- | --- | --- |
|  | Quantile 1 | Quantile 2 | Quantile 3 | Quantile 4 |  |  |
| BADL disability |  |  |  |  |  |  |
| Model 1 | 1.00 (Ref) | 0.66 (0.50, 0.87)^**^ | 0.48 (0.35, 0.65)^***^ | 0.51 (0.38, 0.69)^***^ | ＜0.001 | 0.78 (0.71, 0.86) |
| Model 2 | 1.00 (Ref) | 0.74 (0.55, 0.98)^*^ | 0.55 (0.41, 0.76)^***^ | 0.68 (0.50, 0.92)^*^ | 0.001 | 0.85 (0.77, 0.94) |
| Model 3 | 1.00 (Ref) | 0.76 (0.56, 1.01) | 0.56 (0.40, 0.77)^***^ | 0.67 (0.49, 0.92)^*^ | 0.002 | 0.85 (0.76, 0.94) |
| Model 4 | 1.00 (Ref) | 0.76 (0.57, 1.02) | 0.54 (0.39, 0.75)^***^ | 0.67 (0.49, 0.93)^*^ | 0.002 | 0.85 (0.76, 0.94) |
| IADL disability |  |  |  |  |  |  |
| Model 1 | 1.00 (Ref) | 0.80 (0.67, 0.95)^*^ | 0.53 (0.44, 0.63)^***^ | 0.49 (0.41, 0.59)^***^ | ＜0.001 | 0.77 (0.73, 0.82) |
| Model 2 | 1.00 (Ref) | 0.89 (0.74, 1.07) | 0.59 (0.49, 0.72)^***^ | 0.65 (0.54, 0.80)^***^ | ＜0.001 | 0.84 (0.79, 0.90) |
| Model 3 | 1.00 (Ref) | 0.89 (0.74, 1.08) | 0.59 (0.48, 0.72)^***^ | 0.65 (0.53, 0.80)^***^ | ＜0.001 | 0.84 (0.79, 0.90) |
| Model 4 | 1.00 (Ref) | 0.89 (0.74, 1.08) | 0.58 (0.48, 0.71)^***^ | 0.65 (0.53, 0.80)^***^ | ＜0.001 | 0.84 (0.79, 0.90) |
| ADL disability |  |  |  |  |  |  |
| Model 1 | 1.00 (Ref) | 0.60 (0.45, 0.80)^**^ | 0.41 (0.29, 0.56)^***^ | 0.46 (0.34, 0.63)^***^ | ＜0.001 | 0.74 (0.67, 0.82) |
| Model 2 | 1.00 (Ref) | 0.68 (0.50, 0.91)^*^ | 0.47 (0.34, 0.66)^***^ | 0.62 (0.45, 0.86^)**^ | ＜0.001 | 0.82 (0.73, 0.91) |
| Model 3 | 1.00 (Ref) | 0.71 (0.52, 0.96)^*^ | 0.47 (0.34, 0.67)^***^ | 0.63 (0.45, 0.88)^**^ | ＜0.001 | 0.82 (0.73, 0.91) |
| Model 4 | 1.00 (Ref) | 0.71 (0.52, 0.96)^*^ | 0.46 (0.33, 0.65)^***^ | 0.63 (0.45, 0.88)^**^ | ＜0.001 | 0.82 (0.73, 0.91) |

GNRI, geriatric nutritional risk index; BADL, basic activities of daily living; IADL, instrumental activities of daily living; ADL, activities of daily living; OR,odds ratio; CI, confidence interval.

*, *P*＜0.05; **, *P*＜0.01; *P*＜0.001.

Model 1 was unadjusted model;

Model 2 adjusted for sex, age, marital status, educational attainment, annual income;

Model 3 further adjusted cerebrovascular disease, rheumatism, osteoarthropathy, hypertension, diabetes, estimated glomerular filtration rate, anemia, alanine aminotransferase, aspartate aminotransferase;

Model 4 further adjusted smoking status and alcohol consumption.

Table S10 Association of GNRI (categorized by quartiles) with SIRI, AISI, METS–IR, ABSI, and TyG

| Mediators | Model 1 | |  | Model 2 | |  | Model 3 | |  | Model 4 | |
| --- | --- | --- | --- | --- | --- | --- | --- | --- | --- | --- | --- |
|  | OR (95% CI) | *P* value |  | OR (95% CI) | *P* value |  | OR (95% CI) | *P* value |  | OR (95% CI) | *P* value |
| SIRI | -0.01 (-0.04, 0.02) | 0.509 |  | 0.00 (-0.03, 0.03) | 0.790 |  | -0.01 (-0.04, 0.02) | 0.393 |  | -0.01 (-0.05, 0.02) | 0.373 |
| AISI | -2.55 (-13.57, 8.47) | 0.650 |  | -1.54 (-12.84, 9.77) | 0.790 |  | -3.12 (-14.75, 8.51) | 0.599 |  | -3.36 (-15.06, 8.34) | 0.574 |
| METS–IR | 0.19 (0.17, 0.20) | ＜0.001 |  | 0.20 (0.19, 0.22) | ＜0.001 |  | 0.19 (0.18, 0.21) | ＜0.001 |  | 0.19 (0.18, 0.21) | ＜0.001 |
| ABSI | -0.01 (-0.01, -0.01) | ＜0.001 |  | -0.01 (-0.01, -0.01) | ＜0.001 |  | -0.01 (-0.01, -0.01) | ＜0.001 |  | -0.01 (-0.01, -0.01) | ＜0.001 |
| TyG | 0.11 (0.09, 0.12) | ＜0.001 |  | 0.11 (0.09, 0.12) | ＜0.001 |  | 0.07 (0.06, 0.09) | ＜0.001 |  | 0.07 (0.06, 0.09) | ＜0.001 |

GNRI, geriatric nutritional risk index; SIRI, systemic inflammation response index; AISI, aggregate index of systemic inflammation; METS–IR, metabolic score for insulin resistance; ABSI, A body shape index; TyG, triglyceride–glucose index; OR, odds ratio; CI, confidence interval.

Model 1 was unadjusted model;

Model 2 adjusted for sex, age, marital status, educational attainment, annual income;

Model 3 further adjusted cerebrovascular disease, rheumatism, osteoarthropathy, hypertension, diabetes, estimated glomerular filtration rate, anemia, alanine aminotransferase, aspartate aminotransferase;

Model 4 further adjusted smoking status and alcohol consumption.

Table S11 Association of GNRI with BADL, IADL, and ADL disability by modified Poisson regression analysis

| Models | GNRI, PR (95%CI) | | *P* Value |
| --- | --- | --- | --- |
|  | Normal | Low |  |
| BADL disability |  |  |  |
| Model 1 | 1.00 (Ref.) | 2.37 (1.88, 3.00) | ＜ 0.001 |
| Model 2 | 1.00 (Ref.) | 1.91 (1.52, 2.40) | ＜ 0.001 |
| Model 3 | 1.00 (Ref.) | 1.85 (1.47, 2.33) | ＜ 0.001 |
| Model 4 | 1.00 (Ref.) | 1.85 (1.47, 2.32) | ＜ 0.001 |
| IADL disability |  |  |  |
| Model 1 | 1.00 (Ref.) | 1.57 (1.40, 1.76) | ＜ 0.001 |
| Model 2 | 1.00 (Ref.) | 1.34 (1.20, 1.49) | ＜ 0.001 |
| Model 3 | 1.00 (Ref.) | 1.31 (1.17, 1.47) | ＜ 0.001 |
| Model 4 | 1.00 (Ref.) | 1.32 (1.18, 1.47) | ＜ 0.001 |
| ADL disability |  |  |  |
| Model 1 | 1.00 (Ref.) | 2.56 (2.01, 3.25) | ＜ 0.001 |
| Model 2 | 1.00 (Ref.) | 2.02 (1.59, 2.56) | ＜ 0.001 |
| Model 3 | 1.00 (Ref.) | 1.92 (1.51, 2.44) | ＜ 0.001 |
| Model 4 | 1.00 (Ref.) | 1.91 (1.50, 2.42) | ＜ 0.001 |

GNRI, geriatric nutritional risk index; BADL, basic activities of daily living; IADL, instrumental activities of daily living; ADL, activities of daily living; PR, prevalence ratio; CI, confidence interval.

Model 1 was unadjusted model;

Model 2 adjusted for sex, age, marital status, educational attainment, annual income;

Model 3 further adjusted cerebrovascular disease, rheumatism, osteoarthropathy, hypertension, diabetes, estimated glomerular filtration rate, anemia, alanine aminotransferase, aspartate aminotransferase;

Model 4 further adjusted smoking status and alcohol consumption.

Table S12 Association of SIRI, ABSI, METS–IR, ABSI, and TyG with BADL, IADL, and ADL disability by modified Poisson regression analysis

| Mediators | Model 1 | |  | Model 2 | |  | Model 3 | |  | Model 4 | |
| --- | --- | --- | --- | --- | --- | --- | --- | --- | --- | --- | --- |
|  | PR (95% CI) | *P* value |  | PR (95% CI) | *P* value |  | PR (95% CI) | *P* value |  | PR (95% CI) | *P* value |
| BADL disability | | | | | | | | | | | |
| SIRI | 1.16 (0.96, 1.42) | 0.130 |  | 1.07 (0.88, 1.30) | 0.528 |  | 1.06 (0.87, 1.29) | 0.580 |  | 1.04 (0.86, 1.27) | 0.665 |
| AISI | 1.17 (0.96, 1.43) | 0.114 |  | 1.13 (0.93, 1.37) | 0.230 |  | 1.11 (0.91, 1.35) | 0.305 |  | 1.10 (0.90, 1.33) | 0.353 |
| METS–IR | 1.24 (1.02, 1.52) | 0.031 |  | 1.27 (1.04, 1.55) | 0.018 |  | 1.22 (0.99, 1.50) | 0.065 |  | 1.19 (0.97, 1.46) | 0.100 |
| ABSI | 2.16 (1.74, 2.67) | ＜0.001 |  | 1.94 (1.56, 2.42) | ＜0.001 |  | 1.84 (1.48, 2.29) | ＜0.001 |  | 1.84 (1.48, 2.29) | ＜0.001 |
| TyG | 1.09 (0.90, 1.33) | 0.392 |  | 1.15 (0.95, 1.40) | 0.161 |  | 1.07 (0.86, 1.32) | 0.554 |  | 1.08 (0.87, 1.33) | 0.505 |
| IADL disability | | | | | | | | | | | |
| SIRI | 1.05 (0.96, 1.14) | 0.328 |  | 1.04 (0.95, 1.13) | 0.392 |  | 1.04 (0.96, 1.13) | 0.354 |  | 1.04 (0.96, 1.13) | 0.374 |
| AISI | 1.00 (0.92, 1.10) | 0.922 |  | 0.99 (0.91, 1.07) | 0.744 |  | 0.99 (0.91, 1.08) | 0.821 |  | 0.99 (0.91, 1.08) | 0.810 |
| METS–IR | 0.87 (0.80, 0.95) | 0.002 |  | 0.92 (0.85, 1.00) | 0.054 |  | 0.93 (0.85, 1.01) | 0.079 |  | 0.92 (0.84, 1.00) | 0.050 |
| ABSI | 1.52 (1.39, 1.67) | ＜0.001 |  | 1.27 (1.16, 1.39) | ＜0.001 |  | 1.25 (1.14, 1.37) | ＜0.001 |  | 1.26 (1.15, 1.38) | ＜0.001 |
| TyG | 0.93 (0.85, 1.01) | 0.091 |  | 0.95 (0.88, 1.04) | 0.268 |  | 0.96 (0.88, 1.06) | 0.432 |  | 0.97 (0.89, 1.06) | 0.490 |
| ADL disability | | | | | | | | | | | |
| SIRI | 1.19 (0.97, 1.47) | 0.095 |  | 1.09 (0.89, 1.34) | 0.421 |  | 1.07 (0.87, 1.31) | 0.551 |  | 1.05 (0.86, 1.29) | 0.635 |
| AISI | 1.18 (0.96, 1.46) | 0.110 |  | 1.14 (0.93, 1.40) | 0.222 |  | 1.10 (0.90, 1.35) | 0.350 |  | 1.09 (0.89, 1.34) | 0.401 |
| METS–IR | 1.21 (0.98, 1.49) | 0.072 |  | 1.24 (1.01, 1.53) | 0.040 |  | 1.18 (0.95, 1.47) | 0.133 |  | 1.16 (0.93, 1.44) | 0.195 |
| ABSI | 2.23 (1.78, 2.79) | ＜0.001 |  | 1.96 (1.56, 2.47) | ＜0.001 |  | 1.87 (1.48, 2.36) | ＜0.001 |  | 1.87 (1.48, 2.36) | ＜0.001 |
| TyG | 1.06 (0.86, 1.30) | 0.603 |  | 1.12 (0.91, 1.38) | 0.284 |  | 1.03 (0.82, 1.28) | 0.832 |  | 1.03 (0.83, 1.29) | 0.774 |

SIRI, systemic inflammation response index; AISI, aggregate index of systemic inflammation; METS–IR, metabolic score for insulin resistance; ABSI, A body shape index; TyG, triglyceride–glucose index; BADL, basic activities of daily living; IADL, instrumental activities of daily living; ADL, activities of daily living; PR, prevalence ratio; CI, confidence interval.

Model 1 was unadjusted model;

Model 2 adjusted for sex, age, marital status, educational attainment, annual income;

Model 3 further adjusted cerebrovascular disease, rheumatism, osteoarthropathy, hypertension, diabetes, estimated glomerular filtration rate, anemia, alanine aminotransferase, aspartate aminotransferase;

Model 4 further adjusted smoking status and alcohol consumption.

Table S13 Association of GNRI with BADL, IADL, and ADL disability in population without metabolic syndrome

| Models | GNRI, OR (95%CI) | | *P* value |
| --- | --- | --- | --- |
|  | Normal | Low |  |
| BADL disability |  |  |  |
| Model 1 | 1.00 (Ref.) | 2.74 (2.02, 3.71) | ＜0.001 |
| Model 2 | 1.00 (Ref.) | 2.21 (1.61, 3.02) | ＜0.001 |
| Model 3 | 1.00 (Ref.) | 2.14 (1.55, 2.96) | ＜0.001 |
| Model 4 | 1.00 (Ref.) | 2.16 (1.56, 2.99) | ＜0.001 |
| IADL disability |  |  |  |
| Model 1 | 1.00 (Ref.) | 1.97 (1.59, 2.43) | ＜0.001 |
| Model 2 | 1.00 (Ref.) | 1.62 (1.29, 2.05) | ＜0.001 |
| Model 3 | 1.00 (Ref.) | 1.59 (1.26, 2.02) | ＜0.001 |
| Model 4 | 1.00 (Ref.) | 1.61 (1.27, 2.05) | ＜0.001 |
| ADL disability |  |  |  |
| Model 1 | 1.00 (Ref.) | 2.96 (2.17, 4.03) | ＜0.001 |
| Model 2 | 1.00 (Ref.) | 2.35 (1.71, 3.24) | ＜0.001 |
| Model 3 | 1.00 (Ref.) | 2.25 (1.61, 3.14) | ＜0.001 |
| Model 4 | 1.00 (Ref.) | 2.26 (1.62, 3.16) | ＜0.001 |

GNRI, geriatric nutritional risk index; BADL, basic activities of daily living; IADL, instrumental activities of daily living; ADL, activities of daily living; OR, odds ratio; CI, confidence interval.

Model 1 was unadjusted model;

Model 2 adjusted for sex, age, marital status, educational attainment, annual income;

Model 3 further adjusted cerebrovascular disease, rheumatism, osteoarthropathy, hypertension, diabetes, estimated glomerular filtration rate, anemia, alanine aminotransferase, aspartate aminotransferase;

Model 4 further adjusted smoking status and alcohol consumption.

Table S14 Mediation effects of SIRI, AISI, METS–IR, ABSI, and TyG in the association of GNRI with BADL, IADL, and ADL disability stratified in population without metabolic syndrome

| Mediators | ADE |  |  | ACME |  | PME  (%) |
| --- | --- | --- | --- | --- | --- | --- |
|  | β (95%CI) | *P* value |  | β (95%CI) | *P* value |  |
| BADL disability |  |  |  |  |  |  |
| SIRI | 0.064 (0.036, 0.103) | <0.001 |  | 0.001 (0.000, 0.002) | 0.06 | 1.20 |
| AISI | 0.064 (0.036, 0.094) | <0.001 |  | 0.001 (0.000, 0.002) | 0.08 | 1.34 |
| METS–IR | 0.065 (0.036, 0.096) | <0.001 |  | -0.002 (-0.005, 0.001) | 0.22 | -2.96 |
| ABSI | 0.044 (0.015, 0.068) | <0.001 |  | 0.016 (0.010, 0.022) | <0.001 | 26.32 |
| TyG | 0.064 (0.037, 0.091) | <0.001 |  | -0.001 (-0.003, 0.001) | 0.30 | -1.75 |
| IADL disability |  |  |  |  |  |  |
| SIRI | 0.095 (0.052, 0.136) | <0.001 |  | 0.000 (-0.003, 0.001) | 0.64 | -0.46 |
| AISI | 0.095 (0.044, 0.144) | <0.001 |  | -0.001 (-0.003, 0.001) | 0.36 | -0.96 |
| METS–IR | 0.091 (0.055, 0.143) | <0.001 |  | 0.002 (-0.001, 0.007) | 0.10 | 2.57 |
| ABSI | 0.071 (0.024, 0.112) | <0.001 |  | 0.022 (0.013, 0.033) | <0.001 | 23.96 |
| TyG | 0.094 (0.054, 0.146) | <0.001 |  | -0.001 (-0.005, 0.002) | 0.38 | -1.38 |
| ADL disability |  |  |  |  |  |  |
| SIRI | 0.063 (0.023, 0.088) | <0.001 |  | 0.001 (0.000, 0.002) | 0.16 | 1.13 |
| AISI | 0.063 (0.042, 0.088) | <0.001 |  | 0.001 (0.000, 0.002) | 0.14 | 1.28 |
| METS–IR | 0.064 (0.033, 0.092) | <0.001 |  | -0.002 (-0.004, 0.000) | 0.14 | -3.28 |
| ABSI | 0.043 (0.006, 0.068) | 0.02 |  | 0.016 (0.009, 0.022) | <0.001 | 26.79 |
| TyG | 0.063 (0.025, 0.096) | <0.001 |  | -0.001 (-0.003, 0.001) | 0.62 | -1.28 |

SIRI, systemic inflammation response index; AISI, aggregate index of systemic inflammation; METS–IR, metabolic score for insulin resistance; ABSI, A body shape index; TyG, triglyceride–glucose index; ADE, average direct effects; ACME, average causal mediation effects (indirect effect); PME, proportion of mediation effect; BADL, basic activities of daily living; IADL, instrumental activities of daily living; ADL, activities of daily living; OR, odds ratio; CI, confidence interval.The mediation effects are estimated based on the fully adjusted model, which adjusted for sex, age, marital status, educational attainment, annual income, cerebrovascular disease, rheumatism, osteoarthropathy, hypertension, diabetes, estimated glomerular filtration rate, anemia, alanine aminotransferase, aspartate aminotransferase, smoking status, and alcohol consumption.


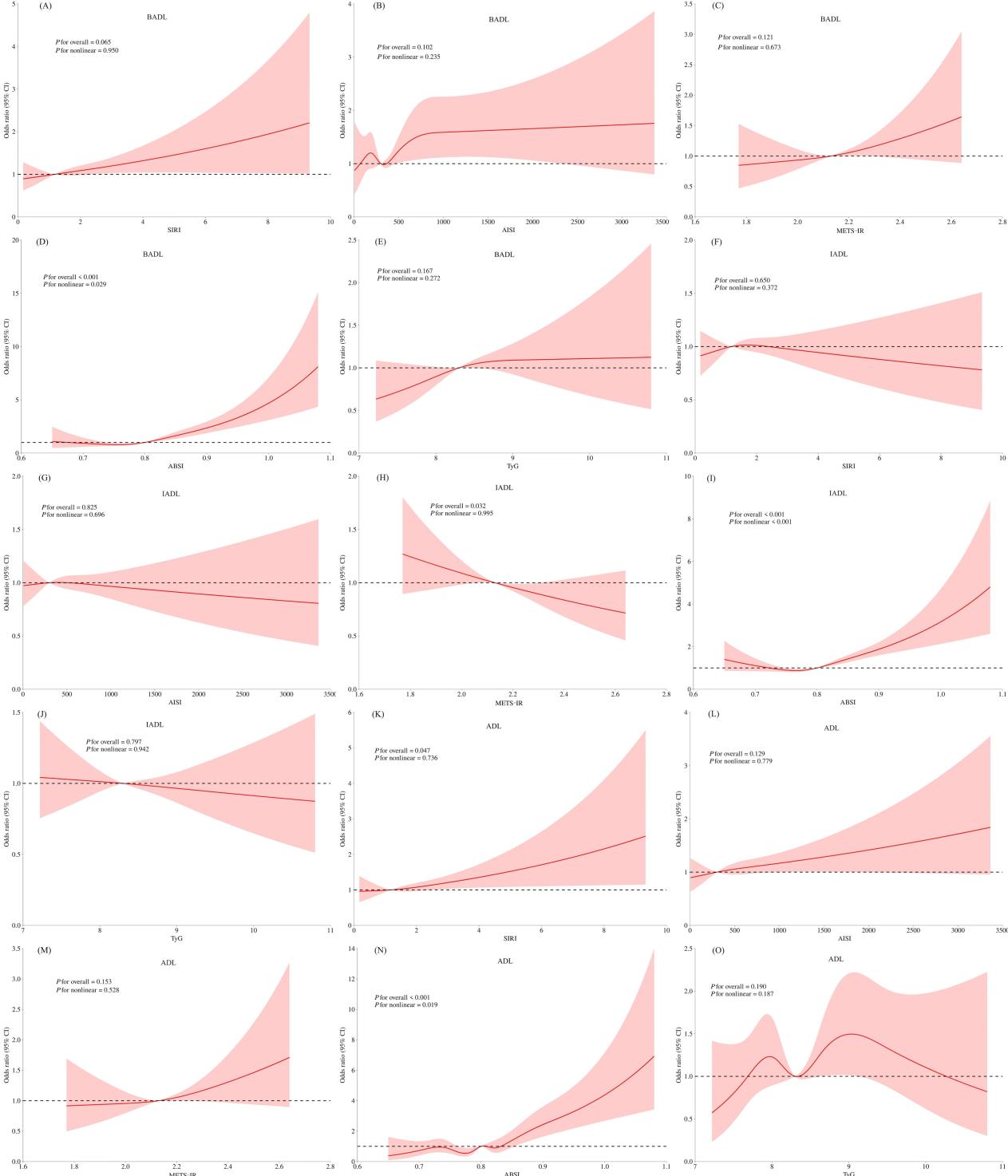


Figure S1 The non–linear relationships of SIRI, AISI, METS–IR, ABSI, and TyG with BADL, IADL, and ADL disability. (A) SIRI with BADL disability, (B) AISI with BADL disability; (C) METS–IR with BADL disability; (D) ABSI with BADL disability; (E) TyG with BADL disability; (F) SIRI with IADL disability; (G) AISI with IADL disability; (H) METS–IR with IADL disability; (I) ABSI with IADL disability; (J) TyG with IADL disability; (K) SIRI with ADL disability; (L) AISI with ADL disability; (M) METS–IR with ADL disability; (N) ABSI with ADL disability; (O)TyG with ADL disability. SIRI, systemic inflammation response index; AISI, aggregate index of systemic inflammation; METS–IR, metabolic score for insulin resistance; ABSI, A body shape index; TyG, triglyceride–glucose index; CI, confidence interval; BADL, basic activities of daily living; IADL, instrumental activities of daily living; ADL, activities of daily living. The models were adjusted for sex, age, marital status, educational attainment, annual income, cerebrovascular disease, rheumatism, osteoarthropathy, hypertension, diabetes, estimated glomerular filtration rate, anemia, alanine, aminotransferase, aspartate aminotransferase, smoking status, and alcohol consumption.


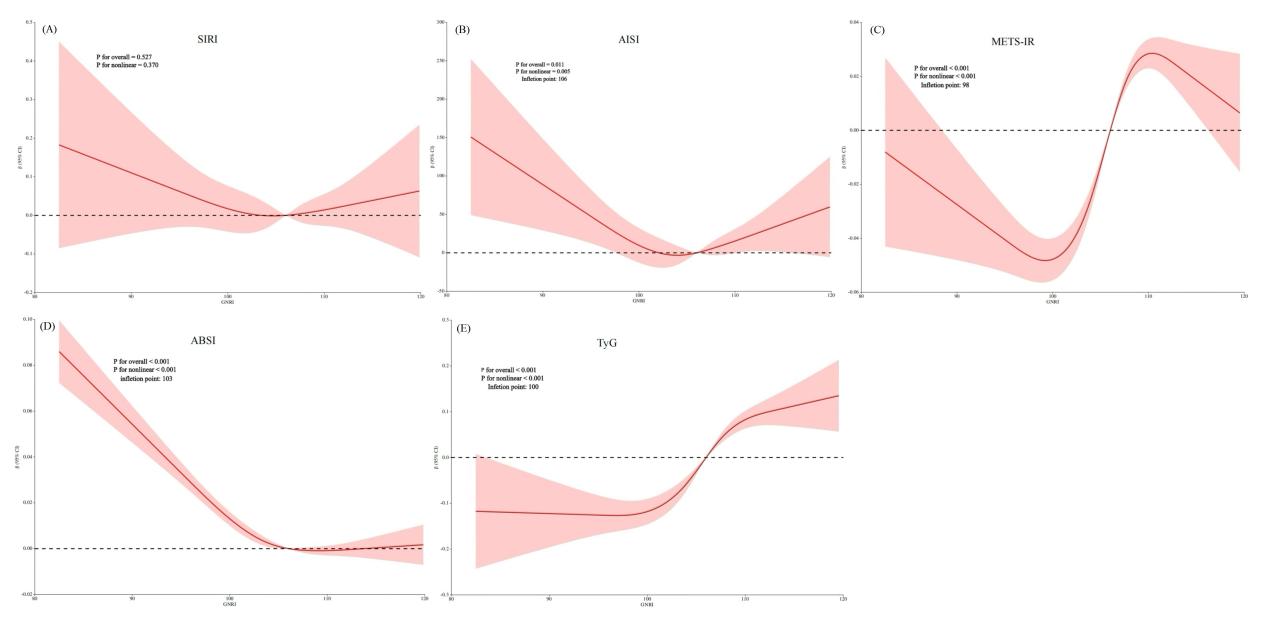


Figure S2 The non–linear relationships of mediators with GNRI. (A) SIRI, (B) AISI, (C) METS–IR, (D) ABSI, (E) TyG. GNRI, geriatric nutritional risk index; SIRI, systemic inflammation response index; AISI, aggregate index of systemic inflammation; METS–IR, metabolic score for insulin resistance; ABSI, A body shape index; TyG, triglyceride–glucose index. The models were adjusted for sex, age, marital status, educational attainment, annual income, cerebrovascular disease, rheumatism, osteoarthropathy, hypertension, diabetes, estimated glomerular filtration rate, anemia, alanine, aminotransferase, aspartate aminotransferase, smoking status, and alcohol consumption.

Figure S3 The non–linear relationships of GNRI with BADL, IADL, and ADL disability in population without metabolic syndrome. (A) BADL disability, (B) IADL disability, (C) ADL disability. GNRI, geriatric nutritional risk index; BADL, basic activities of daily living; IADL, instrumental activities of daily living; ADL, activities of daily living. The models were adjusted for sex, age, marital status, educational attainment, annual income, cerebrovascular disease, rheumatism, osteoarthropathy, hypertension, diabetes, estimated glomerular filtration rate, anemia, alanine, aminotransferase, aspartate aminotransferase, smoking status, and alcohol consumption.
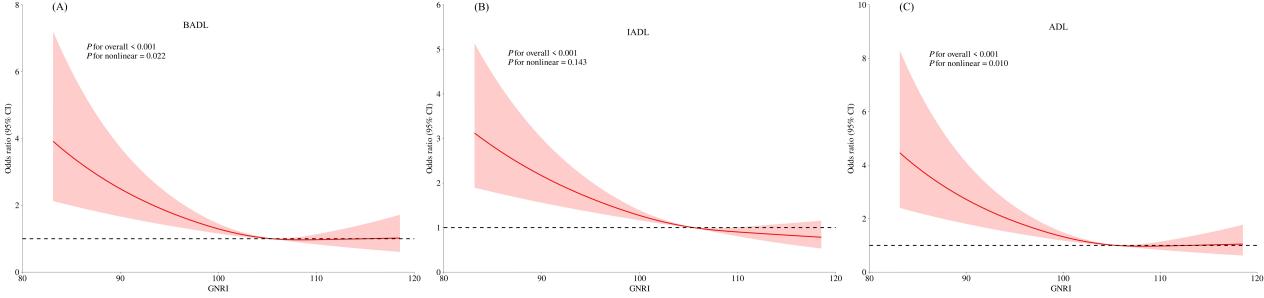


Figure S4 The non–linear relationships of mediators with BADL, IADL, and ADL disability in population without metabolic syndrome. (A) SIRI with BADL disability, (B) AISI with BADL disability; (C) METS–IR with BADL disability; (D) ABSI with BADL disability; (E) TyG with BADL disability; (F) SIRI with IADL disability; (G) AISI with IADL disability; (H) ABSI with IADL disability; (I) METS–IR with IADL disability; (J) ABSI with IADL disability; (K) TyG with IADL disability; (L) SIRI with ADL disability, (M) AISI with ADL disability; (N) METS–IR with ADL disability; (O) ABSI with ADL disability; (P) TyG with ADL disability. SIRI, systemic inflammation response index; AISI, aggregate index of systemic inflammation; METS–IR, metabolic score for insulin resistance; ABSI, A body shape index; TyG, triglyceride–glucose index; CI, confidence interval; BADL, basic activities of daily living; IADL, instrumental activities of daily living; ADL, activities of daily living. The models were adjusted for sex, age, marital status, educational attainment, annual income, cerebrovascular disease, rheumatism, osteoarthropathy, hypertension, diabetes, estimated glomerular filtration rate, anemia, alanine, aminotransferase, aspartate aminotransferase, smoking status, and alcohol consumption.
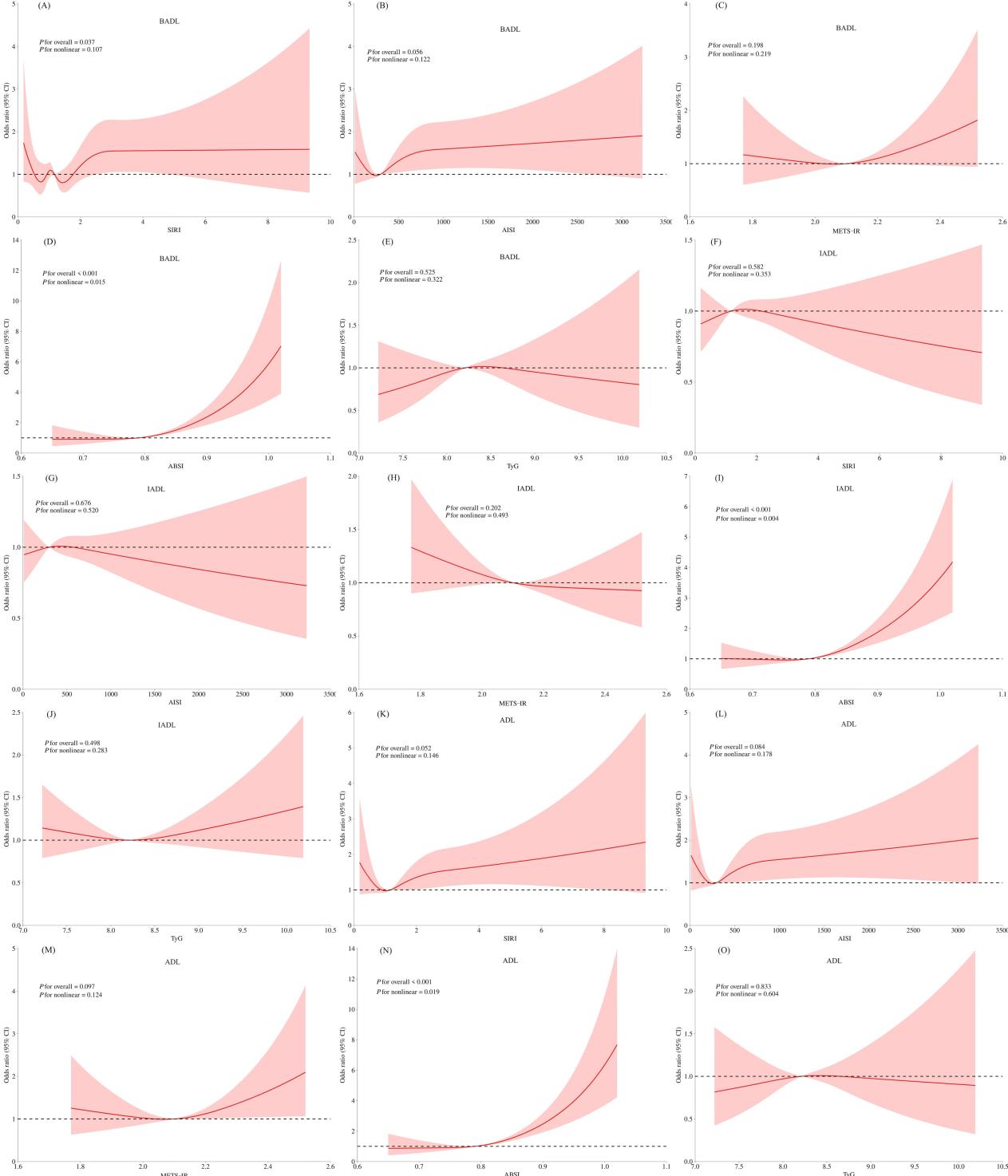

Supplement: Supplementary file 1 [file Supplementary_file_1.DOCX]
